# Identification of metabolites from 2D ^1^H-^13^C HSQC NMR using peak correlation plots

**DOI:** 10.1186/s12859-014-0413-z

**Published:** 2014-12-16

**Authors:** Tommy Öman, May-Britt Tessem, Tone F Bathen, Helena Bertilsson, Anders Angelsen, Mattias Hedenström, Trygve Andreassen

**Affiliations:** Department of Chemistry, Umeå University, Umeå, Sweden; MI Lab, Department of Circulation and Medical Imaging, Norwegian University of Science and Technology (NTNU), Trondheim, Norway; St. Olavs Hospital, Trondheim University Hospital, Trondheim, Norway; Department of Urology, St. Olavs Hospital, Trondheim University Hospital, Trondheim, Norway; Department of Cancer Research and Molecular Medicine, NTNU, Trondheim, Norway; MR Core Facility, Department of Circulation and Medical Imaging, NTNU, Trondheim, Norway

**Keywords:** HSQC, Correlation, Metabolite, Biofluid, Identification

## Abstract

**Background:**

Identification of individual components in complex mixtures is an important and sometimes daunting task in several research areas like metabolomics and natural product studies. NMR spectroscopy is an excellent technique for analysis of mixtures of organic compounds and gives a detailed chemical fingerprint of most individual components above the detection limit. For the identification of individual metabolites in metabolomics, correlation or covariance between peaks in ^1^H NMR spectra has previously been successfully employed. Similar correlation of 2D ^1^H-^13^C Heteronuclear Single Quantum Correlation spectra was recently applied to investigate the structure of heparine. In this paper, we demonstrate how a similar approach can be used to identify metabolites in human biofluids (post-prostatic palpation urine).

**Results:**

From 50 ^1^H-^13^C Heteronuclear Single Quantum Correlation spectra, 23 correlation plots resembling pure metabolites were constructed. The identities of these metabolites were confirmed by comparing the correlation plots with reported NMR data, mostly from the Human Metabolome Database.

**Conclusions:**

Correlation plots prepared by statistically correlating ^1^H-^13^C Heteronuclear Single Quantum Correlation spectra from human biofluids provide unambiguous identification of metabolites. The correlation plots highlight cross-peaks belonging to each individual compound, not limited by long-range magnetization transfer as conventional NMR experiments.

**Electronic supplementary material:**

The online version of this article (doi:10.1186/s12859-014-0413-z) contains supplementary material, which is available to authorized users.

## Background

NMR (nuclear magnetic resonance) spectroscopy is well suited for analysis of complex mixtures of organic compounds and has some distinct advantages compared to other analytical techniques such as GC-MS (gas chromatography–mass spectrometry) and LC-MS (liquid chromatography-mass spectrometry). Most important, NMR spectroscopy is highly reproducible, does not require any sample derivatization and gives detailed structural information about the components of a mixture. The drawback of NMR spectroscopy is the inherent low sensitivity compared to MS-based methods, but it has nevertheless become a cornerstone in metabolomic studies [1].

A vast majority of NMR-based metabolomics studies have been based on 1D ^1^H NMR experiments because of the high sensitivity of the ^1^H nucleus. Recent technical advances with higher magnetic fields and the introduction of cryogenic probes have drastically increased the sensitivity and thereby reduced experimental times for inverse detection experiments of other nuclei such as ^13^C and ^31^P. This allows analyses of large data sets of dilute samples, e.g. biofluids, within a reasonable timeframe. Heteronuclear 2D NMR methods provide additional structural information and are important tools for structure elucidation of new compounds. There are a number of inverse heteronuclear 2D NMR experiments available, and the two most important are Heteronuclear Single Quantum Correlation (HSQC) and Heteronuclear Multiple-Bond Correlation (HMBC). HSQC spectra reveal the chemical shifts of ^1^H and X-nuclei directly bonded to each other, whereas HMBC spectra reveal correlations over multiple bonds (typically 2–3). Especially, the ^1^H-^13^C HSQC experiment has had a pivotal role in organic chemistry. In addition to being a relatively sensitive experiment, the large chemical shift range for ^13^C in a ^1^H-^13^C HSQC spectrum reduces spectral overlap which greatly benefits compound identification. Compared to 1D ^1^H NMR spectra, the ^1^H-^13^C HSQC spectrum provides a more detailed biochemical fingerprint, which has recently spurred interest in HSQC based metabolic profiling and multivariate analysis of human biofluids [2,3].

In order to draw biologically relevant conclusions from metabolomics studies, identification of key metabolites is required. This can be challenging considering the vast amount of metabolites present in biological samples such as human biofluids, extracts of plants or cell cultures which results in many overlapping peaks in the NMR spectra [4]. For ^1^H NMR this has partly been resolved by fitting the experimental spectra to simulated or experimentally obtained spectra from single metabolites [5]. Another interesting approach to identify metabolites is by Statistical Total Correlation Spectroscopy, STOCSY [6,7], which utilizes statistical correlation between peaks throughout a series of spectra. Peaks that vary in intensities in a highly correlated manner are likely to belong to the same compound. Correlations may also be observed between related compounds, e.g. metabolites belonging to the same biological pathway, but such intermolecular correlations should always be weaker than intramolecular ones. Together with established multivariate methods such as principal component analysis, PCA [8,9] or orthogonal projections to latent structures, OPLS [10], this approach can be used to identify metabolites that vary between different classes of samples. Since STOCSY was first reported, a number of related tools have emerged which are useful for metabolic pathway analysis as well as biomarker identification [11]. A common denominator for these tools is that they exploit the statistical correlation between spectral data from *multiple* biological mixtures. On the contrary, a number of tools for statistical correlation of NMR data recorded from a *single* sample have also been developed. These tools include covariance NMR [12], indirect covariance NMR [13] and higher-rank correlation NMR [14]. Covariance NMR is an alternative to traditional 2D Fourier transformation of homonuclear 2D spectra like Total Correlation Spectroscopy (TOCSY) and Nuclear Overhauser Effect Spectroscopy (NOESY). By correlating the data along the indirect dimension, highly resolved 2D correlation plots can be produced with fewer t1 increments as compared to using standard 2D Fourier transformation. Indirect covariance NMR uses the same principles to generate 2D pseudospectra from more easily obtained spectra, like ^13^C-^13^C correlation spectra from ^1^H-^13^C HSQC-TOCSY [13] or from a combination of ^1^H-^1^H Correlation Spectroscopy (COSY) and ^1^H-^13^C HSQC [15]. Higher-rank correlation NMR takes it one step further, correlating 2D NMR data from two or more sources, forming 3D or higher dimensional correlation spectra. An example of this method, and relevant to the work presented in this paper, is the merging of ^1^H-^13^C HSQC and 2D ^1^H-^13^C HSQC-TOCSY spectra to form a triple rank (3R) HSQC-TOCSY spectrum [16]. From this spectrum, HSQC spectra of individual mixture components may be extracted, providing that the involved protons belong to the same spin system. If a compound consists of multiple isolated spin systems, these correlation methods will fail to reveal all associated peaks. This is not the case for the STOCSY-like methods, since correlations do not depend on any spin-spin couplings across multiple bonds.

In STOCSY, peaks which originate from the same compound should correlate perfectly, but overlapping peaks from several metabolites in crowded regions of ^1^H spectra will, however, have a negative impact on the correlation. This may preclude the detection of important resonances from key metabolites. In a recent paper by Rudd *et al.*, a STOCSY-like correlation method using 2D HSQC instead of 1D ^1^H NMR data was presented [17]. This method, termed HSQCcos, was used to extract structural information from different compositions of the heterogeneous polysaccharide heparine. Contemporary with Rudd, we have worked on correlating HSQC spectra from post-prostatic palpation urine. The aim of this paper is to demonstrate that the method can be used for unambiguous metabolite identification in biofluids. With increased use of HSQC data in multivariate analysis, we envision that the HSQCcos method will become a valuable asset for interpretation of multivariate models.

## Methods

### Sample preparation and NMR analyses

The study was approved by The Regional Committee for Medical and Health Research Ethics (Norwegian Health Region III) and informed written consent was obtained from all 50 patients.

The 50 frozen (−80°C) urine samples from 50 different patients, collected after transrectal palpation of the prostate (three strokes over each lobe), were thawed at room-temperature for 20 minutes. Each sample (1 ml) was spun at 13000 g for 5 min and 540 μl of the supernatant was mixed with 60 μl D_2_O containing PBS buffer and TSP-d_4_, resulting in a total volume of 600 μl. The samples were vortexed and transferred to 5 mm NMR-tubes (Bruker Biospin, Rheinstetten, Germany) before analysis. The spectra were acquired using a Bruker Avance III 600 MHz spectrometer, equipped with a QCI cryoprobe. A Bruker SampleJet and ICON-NMR software (Bruker Biospin) were used to record all spectra automatically. The spectra were obtained at a constant temperature 300 K using the HSQC (hsqcetgpsisp.2) pulse sequence with 256 increments, 16 transients, a 1 s relaxation delay, sweep widths of 16 and 165 ppm and offset 4.7 and 75 ppm for the ^1^H and ^13^C dimension, respectively. The sequence was optimized for direct coupling constants of 145 Hz, which is a common compromise between aliphatic and aromatic signals. Total acquisition time for each experiment was 77.5 minutes. The data were processed with Topspin 3.2 (Bruker Biospin) using a 90° shifted qsine window function to a total of 1024 × 512 data points (F2 × F1), followed by automated baseline- and phase correction.

All spectra were calibrated relative to the TSP peak in both dimensions. Most of the metabolites were identified by comparison with reference spectra from the Human Metabolome Database (HMDB) [18].

### Correlation analyses

Statistical Total Correlation Spectroscopy performed on ^1^H spectral data is based on equation 1, where **C** is the correlation matrix, n is the number of spectra and **X** is the autoscaled and mean-centered matrix of the spectra with size n x K where K is number of variables (data points) in the spectra.

1 $$ \mathbf{C} = \frac{1}{n-1}{\mathbf{X}}^{\mathrm{t}}\mathbf{X} $$

In this study we opted for an alternative approach, where instead of calculating the complete correlation matrix, one peak of interest, **v**_peak_, is chosen and only correlations to that peak are calculated (equation 2). Thus, **c**_peak_ will in this case be a vector from which a 2D correlation plot is constructed.

2 $$ {\mathbf{c}}_{\mathrm{peak}}=\frac{1}{n-1}{{\mathbf{v}}_{\mathrm{peak}}}^{\mathrm{t}}\mathbf{X} $$

**Figure 1:**
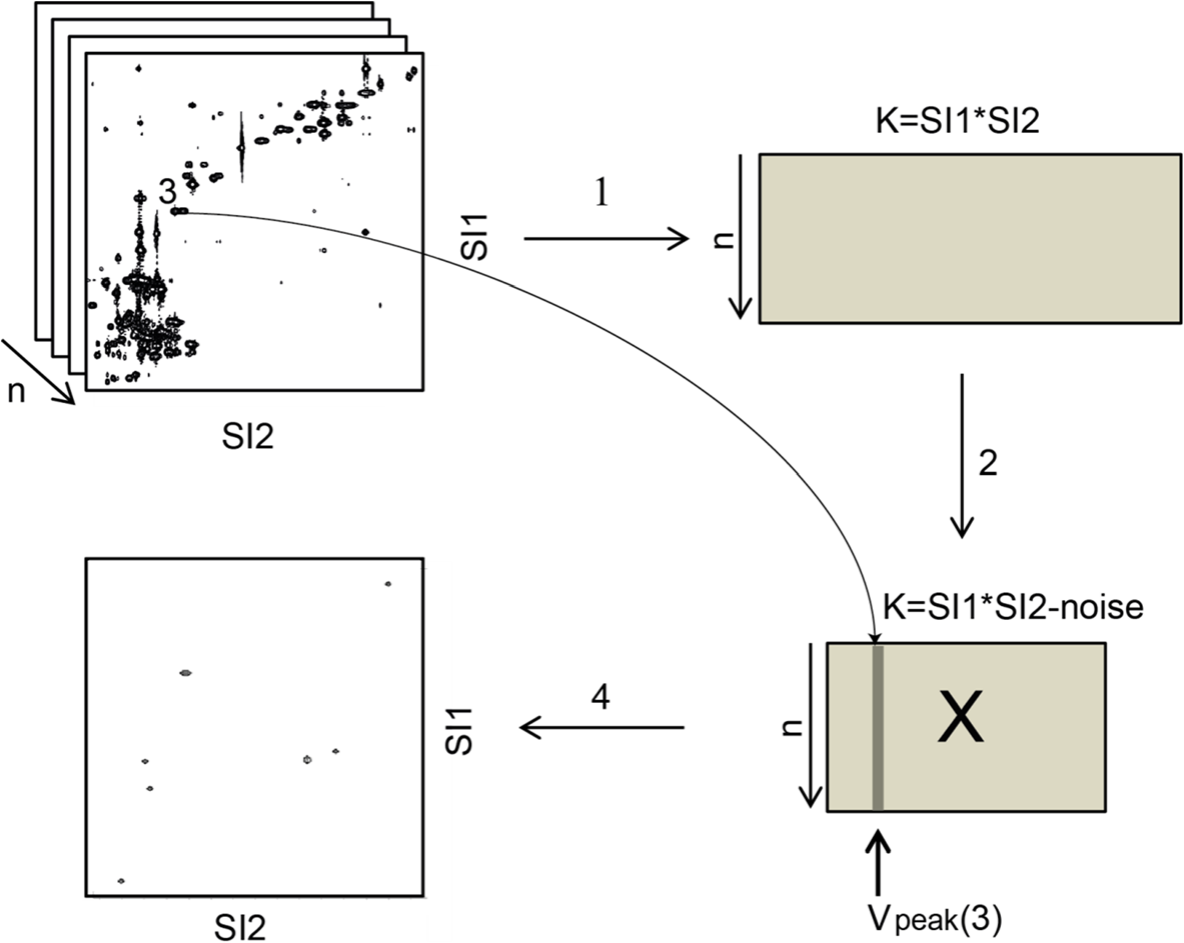
**Procedure for generating correlation plots.** Each spectrum is transformed to a row vector where the chemical shifts for both ^1^H and ^13^C are encoded, forming a matrix with dimensions n x K (step 1). By plotting one of these vectors, real signals are easily discerned from noise and an appropriate noise threshold may be selected. Data points are removed from the matrix only when all values in the column (from all HSQC spectra) are lower than the selected threshold. This noise exclusion step results in a final matrix **X** of a more manageable size that still contains all relevant information (step 2). Any of the rows in **X** can be transformed to a matrix of the original format and plotted as a noise-free HSQC-spectrum. From this plot, a cross-peak (coordinate) of interest may be selected, corresponding to the column vector **v**_peak_ (step 3) in **X**. At this point, **X** (and **v**_peak_) is auto-scaled and a correlation vector **c**_peak_ is calculated according to equation 2. This vector will contain values between −1 and 1, i.e. correlation coefficients, and can be visualized as a 2D spectrum after re-introducing zeros to the data points omitted in the noise exclusion step, followed by transformation to a matrix with the same dimensions as the original data (step 4). A cutoff for the correlation is then chosen for the visualization, for example 0.9, to only show peaks highly correlated (>0.9) with the chosen peak.

This approach is similar to the one used by Rudd et al. [17]. The peaks of interest were selected in a point-and-click fashion from a plot of a representative HSQC spectrum. Each HSQC cross-peak encompasses a number of data points, and to remedy small changes in chemical shift, the most central data point within each cross-peak was selected. This usually coincided with the local maxima. The correlation coefficients calculated range from −1 to 1, with 1 meaning perfect positive correlation. By only plotting the most highly correlated data points, i.e. setting a high cutoff for the correlation coefficient, HSQC spectra of seemingly pure compounds could be produced. A pictorial overview of the procedure is presented in Figure 1, starting from aligned and normalized (optional) ^1^H-^13^C HSQC spectra. All steps, including alignment and normalization, have been implemented in Matlab (Mathworks, Natick, MA) scripts together with a graphical user interface developed in-house. The scripts import ^1^H-^13^C HSQC spectra in Bruker format (2rr files) and can also export the resulting correlation plots in Bruker format for visualization in Topspin. All functions are activated from an intuitive graphical interface, making them easily accessible for unexperienced Matlab users. Matlab scripts are available upon request.

## Results and discussion

**Figure 2:**
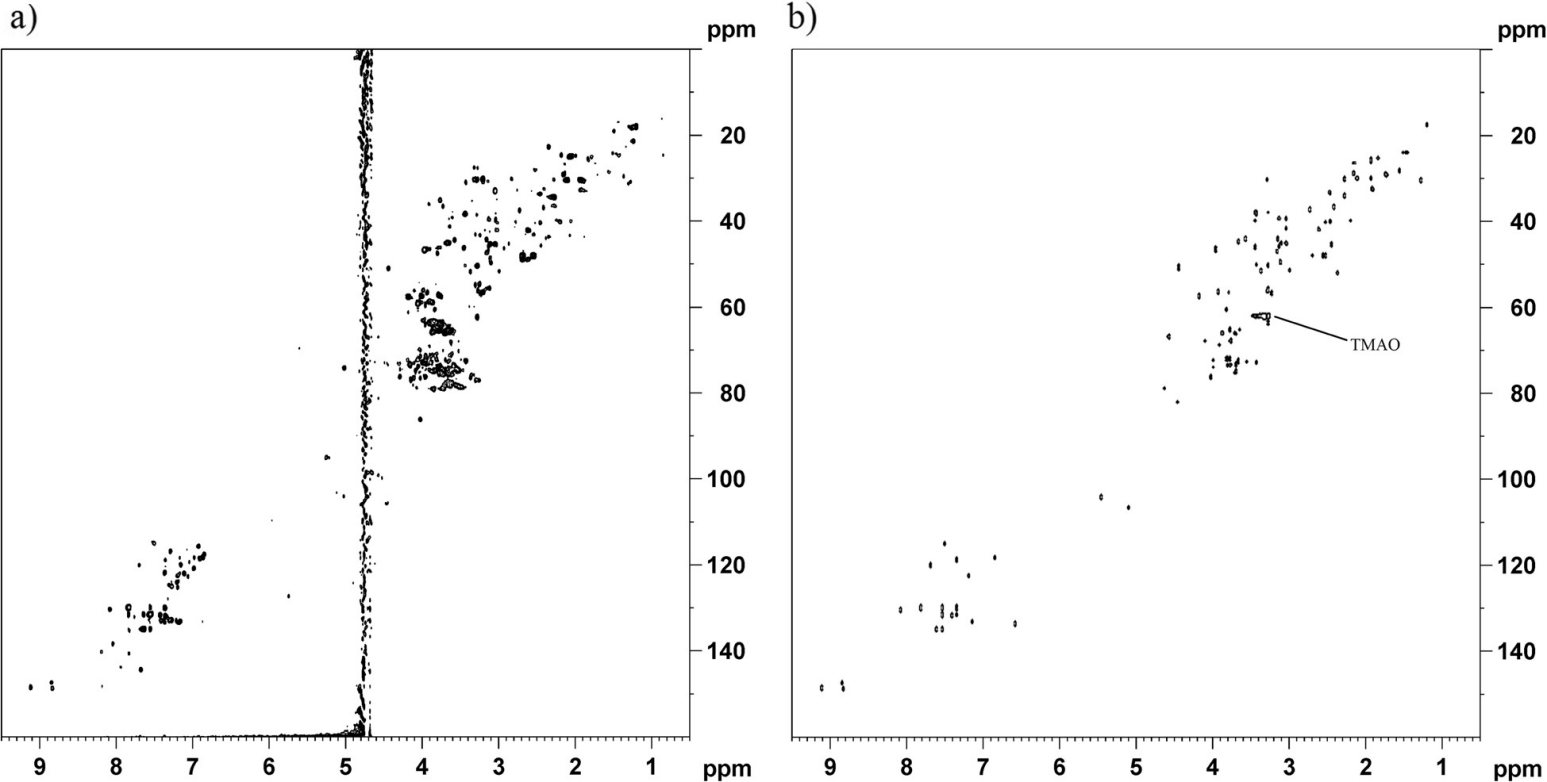
**Real and constructed** ^**1**^ **H-** ^**13**^ **C HSQC spectra from post-prostatic palpation urine.** To the left **(a)** is one of the 50 recorded ^1^H-^13^C HSQC spectra from post-prostatic palpation urine. To the right **(b)** is a constructed ^1^H-^13^C HSQC spectrum, prepared by merging correlation plots from 23 metabolites. Peaks from 7 metabolites with only one ^1^H-^13^C HSQC cross-peak are also included. TMAO appears broad due to phase distortion.

A representative ^1^H-^13^C HSQC spectrum from post-prostatic palpation urine is shown in Figure 2a. The Human Metabolite Database (HMDB) [18] was browsed for urinary metabolites with expected high levels (above 20 μmol/mmol creatinine). When HSQC data was available, correlation plots were produced selecting one of the cross-peaks from the metabolites in question. The Pearson correlation coefficients calculated range from −1 to 1, with 1 meaning a perfect positive correlation. To generate clean plots, only the most highly correlated peaks were shown. In many cases, a cutoff value of 0.9 provided perfect correlation plots, only containing the cross-peaks as expected from the reference. In other cases, some fine tuning of the cutoff was required before a satisfactory plot could be produced. In addition to typical urinary metabolites, post-prostatic palpation urine contains metabolites originating from the prostate. One of these is spermine, which is included in the list of 23 metabolites unambiguously identified by their correlation plots (Table 1).

**Table 1:** **Identified metabolites from post-prostatic palpation urine**

**Metabolite**	**Selected peak (** ^**1**^ **H/** ^**13**^ **C) [ppm]**	**Cutoff**	**Number of correlating peaks (found/expected)**
Trigonelline	9.12 / 148.4	0.9	5 / 5
Hippuric acid	7.82 / 129.6	0.9	4 / 4
Indoxyl sulphate	7.69 / 119.9	0.8	4 / 5
Phenylacetylglutamine^*^	7.41 / 131.5	0.8	8 / 8
*trans*-Aconitic acid	6.58 / 133.5	0.74	3 / 2
Levoglucosan	5.45 / 104.0	0.883	9 / 7
Carnitine^**^	4.56 / 66.8	0.7	4 / 4
Creatine	3.92 / 56.5	0.9	2 / 2
Mannitol	3.80 / 72.0	0.9	4 / 4
Erythritol	3.69 / 74.9	0.867	3 / 3
Galactitol	3.66 / 72.6	0.65	3 / 3
Glycine	3.56 / 44.2	0.8	1 / 1
Taurine	3.44 / 38.0	0.9	2 / 2
4-Hydroxyphenylacetic acid	3.44 / 46.1	0.9	3 / 3
Methanol	3.36 / 51.6	0.8	1 / 1
1-Methyluric acid	3.28 / 30.3	0.8	1 / 1
Betaine	3.26 / 55.8	0.8	2 / 2
TMAO	3.26 / 62.0	0.8	1 / 1
Ethanolamine	3.14 / 44.2	0.85	2 / 2
Isocitric acid	2.98 / 51.6	0.8476	5 / 4
Dimethylamine	2.72 / 37.4	0.8	1 / 1
Citric acid	2.54 / 48.1	0.899	2 / 2
Succinic acid	2.40 / 36.7	0.8	1 / 1
Glutamine	2.14 / 29.0	0.8	3 / 3
Acetic acid	1.92 / 26.1	0.8	1 / 1
Spermine	1.81 / 25.4	0.9	5 / 5
Lysine	1.70 / 29.0	0.7	5 / 6
Adipic acid	1.54 / 28.3	0.83	2 / 2
3-Hydroxyisovaleric acid	1.26 / 30.6	0.8	2 / 2
3-Aminoisobutanoic acid	1.20 / 17.6	0.8	4 / 4

*NMR data not reported in HMDB. Correlating ^1^H signals compatible with literature values [19].

**HSQC data not available in HMDB, compared to data from YMDB [20].

**Figure 3:**
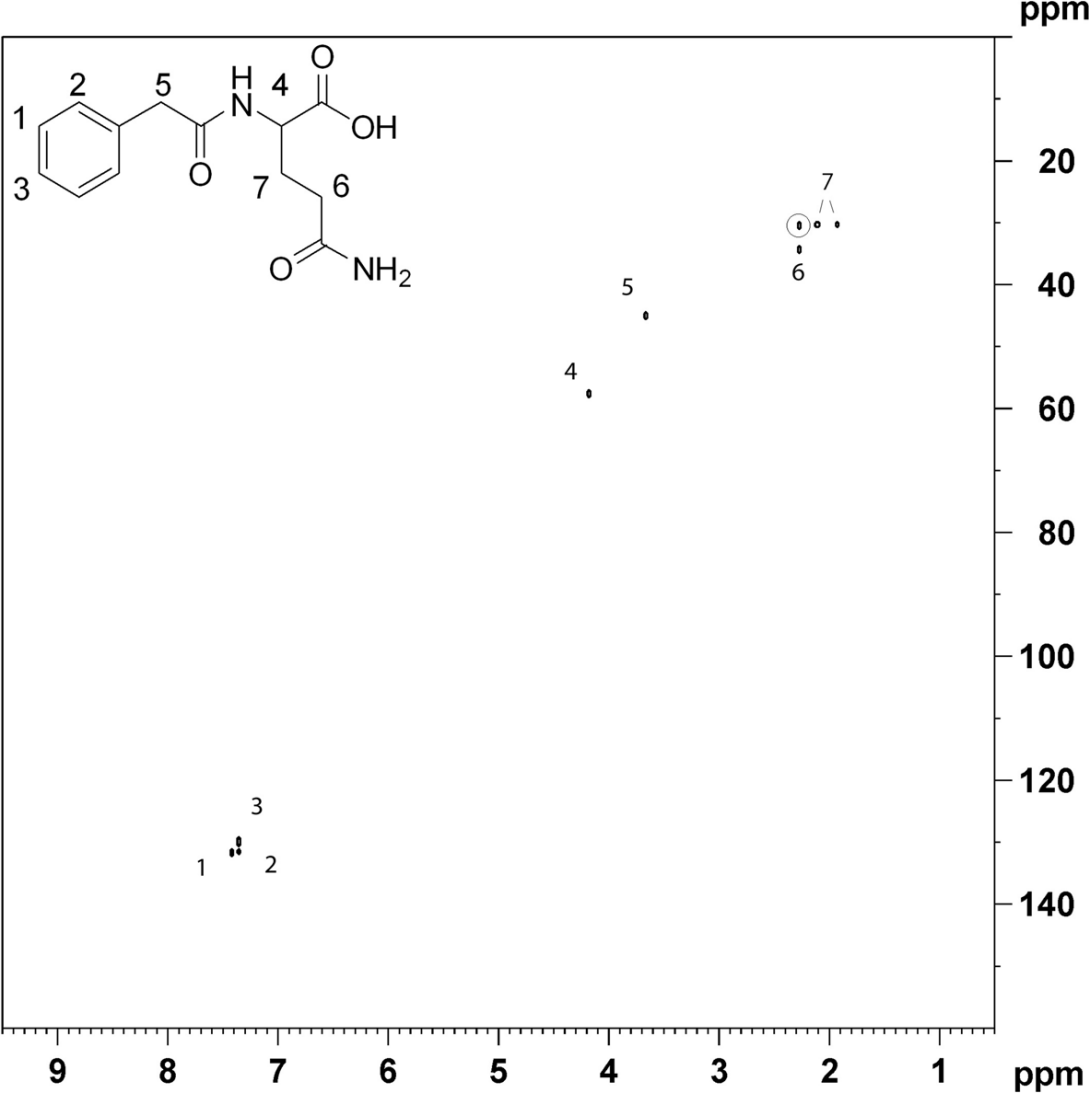
**HSQC correlation plot of phenylacetylglutamine.** Correlation plot showing all data points correlating strongly with 7.41 / 131.5 ppm (^1^H / ^13^C) (correlation coefficient higher than 0.8). Correlation to long-range cross-peak is circled. Two cross-peaks are marked with “7” due to diastereotopic protons in this position.

Some of the plots contained unexpected additional cross-peaks (found peaks > expected peaks), possibly because of correlation with some unknown metabolite due to similar biological regulation. Other plots had missing correlations, as expected when certain cross-peaks fall into regions with heavy overlap. The presence of phenylacetylglycine in human urine is controversial, with some groups claiming to have identified it by NMR [21], and others claiming it cannot be detected by GC-MS [22]. If NMR-based identification of phenylacetylglycine is based on signals from the benzyl group, it is likely to be mistaken with phenylacetylglutamine, which contains a similar group with overlapping signals. Creating a correlation plot from one of these signals clearly shows cross-peaks indicative of phenylacetylglutamine, and no sign of the expected phenylacetylglycine signal at 3.74/46.2 ppm (^1^H / ^13^C) (Figure 3). No ^13^C NMR data of phenylacetylglutamine could be found from literature, but ^1^H NMR data is compatible with reported values [19]. Although we cannot disproof small amounts of phenylacetylglycine by our method, it is obvious that phenylacetylglutamine is the dominating of the two in our study. The example also demonstrates how statistical correlation can connect signals from isolated spin systems (benzyl part and amino acid part), not depending on weak/impossible long-range magnetization transfer. This is in contrast to triple rank correlation NMR which is purely based on spin-spin correlation [16].

Although HSQC experiments are optimized for direct coupling ^1^ J ^1^H-^13^C of 145 Hz, long-range cross-peaks due to large ^2^ J or ^3^ J couplings can often be seen. These peaks are present in the original spectra at low intensities, but appear clearly in the correlation plots as they are just as highly correlated to the chosen peak as the peaks from ^1^ J ^1^H-^13^C couplings. These peaks resemble what you would expect to see in a ^1^H-^13^C HMBC spectrum and actually provide additional information that could benefit structural assignment. One example of such long-range cross-peak is 2.27 / 30.4 ppm (^1^H / ^13^C) as noted in Figure 3 for phenylacetylglutamine. Naturally, for metabolites at low concentration, these peaks fall below the detection limit.

Merging all the produced correlation plots gives the combined spectrum shown in Figure 2b. This spectrum also includes peaks from 7 metabolites with only one HSQC cross-peak, namely acetic acid, dimethylamine, glycine, methanol, 1-methyluric acid, succinic acid, and trimethylamine N-oxide (TMAO). These are all expected urine metabolites and their cross-peaks did not correlate with any other peaks (with correlation coefficient >0.8). Correlation plots of each individual metabolite are available in Additional file 1.

Not all cross-peaks may be accounted for, but the combined spectrum shows clear resemblance to the real HSQC spectrum in Figure 2a. Each HSQC cross-peak is usually defined by more than one data point, meaning that each data point or coordinate is likely to correlate very well with one or more of its neighbors. This explains why some peaks in Figure 2b appear broader than others, including the signal from (TMAO) at 3.26 / 62.0 ppm (^1^H / ^13^C) which is slightly phase distorted in some of the recorded HSQC spectra. Correlation to such clusters of data points can prove beneficial in cases where the number of recorded spectra is low, clearly distinguishing correlation to real cross-peaks from coincidentally correlating data points (e. g. regions with much overlapping signals).

In biofluids, and especially in urine samples, chemical shift variation can be substantial due to differences in ionic strength and pH. However, the current result shows that spectra from challenging and complex biofluids can be used to create HSQC correlation plots, without need for any peak alignment algorithm. However, in extreme cases chemical shift variation will result in low correlation between peaks belonging to the same compound. Peak alignment tools like icoshift [23] adapted to HSQC-spectra might remedy this. However, our results show that small deviation of chemical shifts is tolerable and the robustness of the method is demonstrated by using non-peak aligned spectra.

Selecting only one data point within each peak to create correlation plots proved very satisfactory. However, the method could be further expanded by selecting multiple data points for each cross-peak (e.g. all points within predefined ^1^H and ^13^C NMR chemical shift ranges), generating multiple correlation plots that could be merged into one. For this merged correlation plot we should expect more clusters of actually correlated cross-peaks, distinguishing them from coincidentally correlating data points.

Structure elucidation by an HSQC spectrum alone is a difficult task since it lacks the necessary long range couplings needed to identify extended spin systems. Regardless, HSQC spectra of individual metabolites represent useful fingerprints for structure confirmation, especially with more reference spectra like those from HMDB becoming available. When real reference spectra are not available, the HSQC-correlation plots may be compared to calculated spectra from quantum mechanically based NMR prediction software. In principle, similar correlation plots could be produced from other 2D NMR spectra like COSY, TOCSY or HMBC. If sample integrity is preserved during acquisition, metabolite variation should be identical within each type if 2D spectrum. This implies that a selected HSQC cross-peak not only correlates with other HSQC cross-peaks belonging to the same compound, but also e.g. the corresponding COSY cross-peaks. Combining 2D NMR spectra this way constitutes a powerful tool for the elucidation of novel compounds without tedious and often difficult chromatographic separation.

## Conclusions

In this paper, we have shown how covariance analysis of 2D ^1^H-^13^C HSQC spectra can be used to create sub-spectra from individual metabolites in complex human biofluids. These sub-spectra are derived from the variation in metabolic composition within a series of spectra and do not depend on long–range magnetization transfer between spins. As a result, HSQC cross-peaks from isolated spin-system, separated by magnetically silent regions, are effectively displayed in the same plot. From the post-prostatic palpation urine spectra, 23 metabolites were easily identified by their sub-spectra. The results demonstrate that HSQCcos in general is a useful tool for identifying key metabolites in biofluids, producing HSQC-spectra resembling pure compounds without chromatographic separation. These spectra provide useful fingerprints for database queries. If combined with similar analyses of additional 2D NMR datasets such as COSY and/or TOCSY, complete structure elucidation could be achieved without isolating the individual components.

### Availability of supporting data

The data set supporting the results of this article is included within the article and its additional files.

## Additional file

Additional file 1:
**Correlation_plots.** Shows individual correlation plots from all 23 metabolites.
