# Supplementary material for: Identification of metabolites from 2D 1H-13C HSQC NMR using peak correlation plots
Source: BMC Bioinformatics. 2014 Dec 16;15(1):413. doi: 10.1186/s12859-014-0413-z (PMC4274720; doi:10.1186/s12859-014-0413-z)

Trigonelline

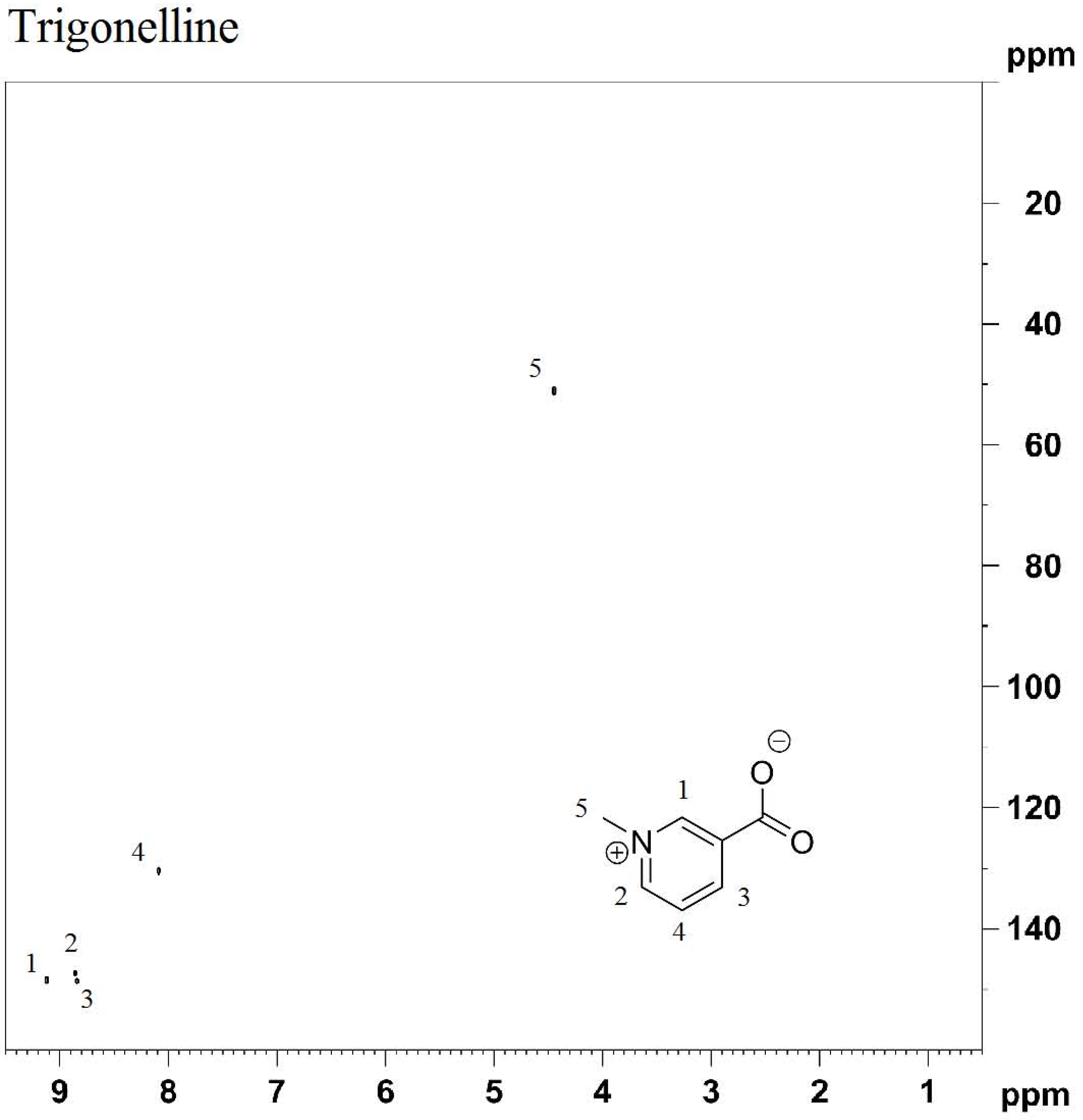


Hippuric acid

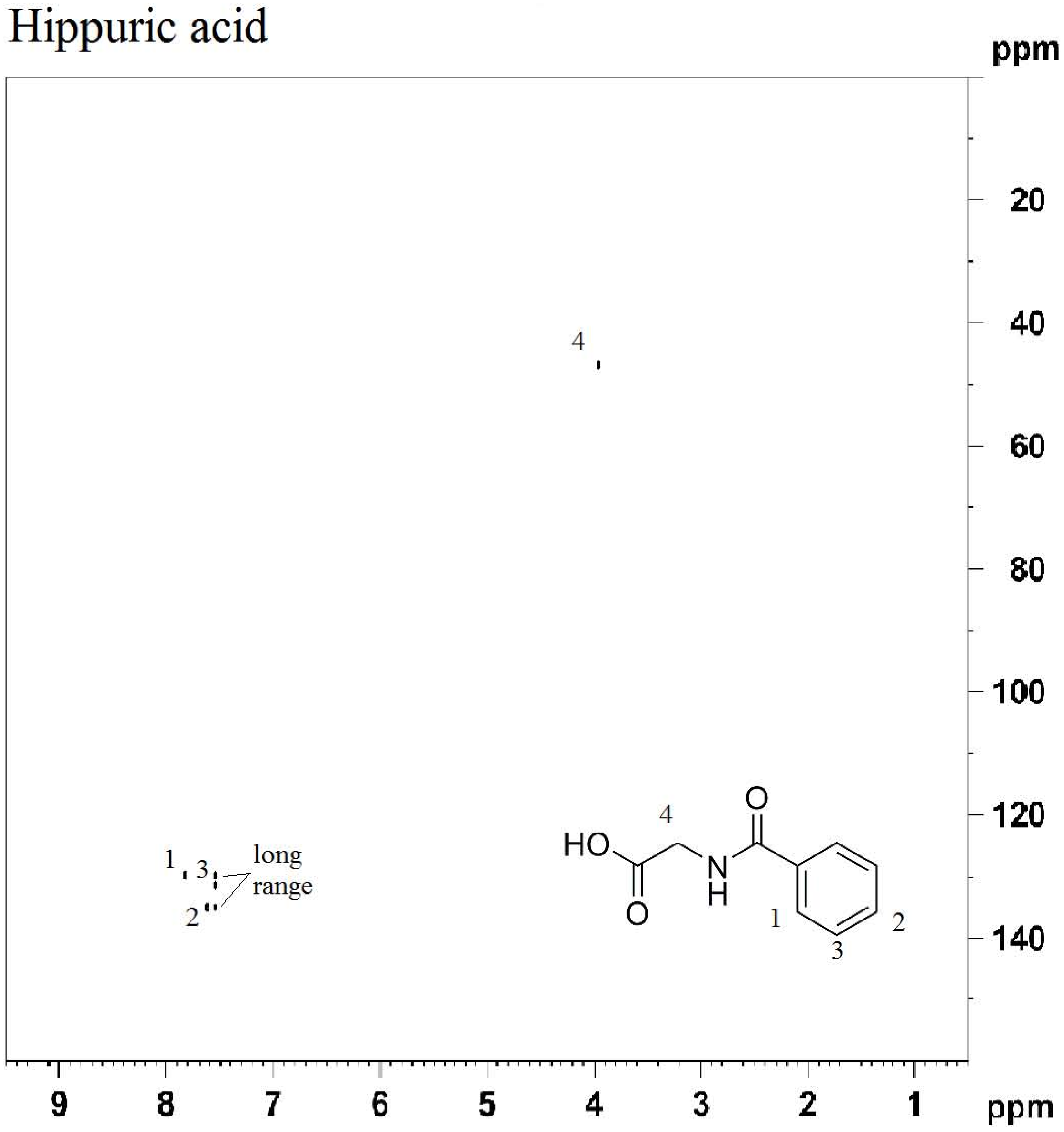


Indoxyl sulfate

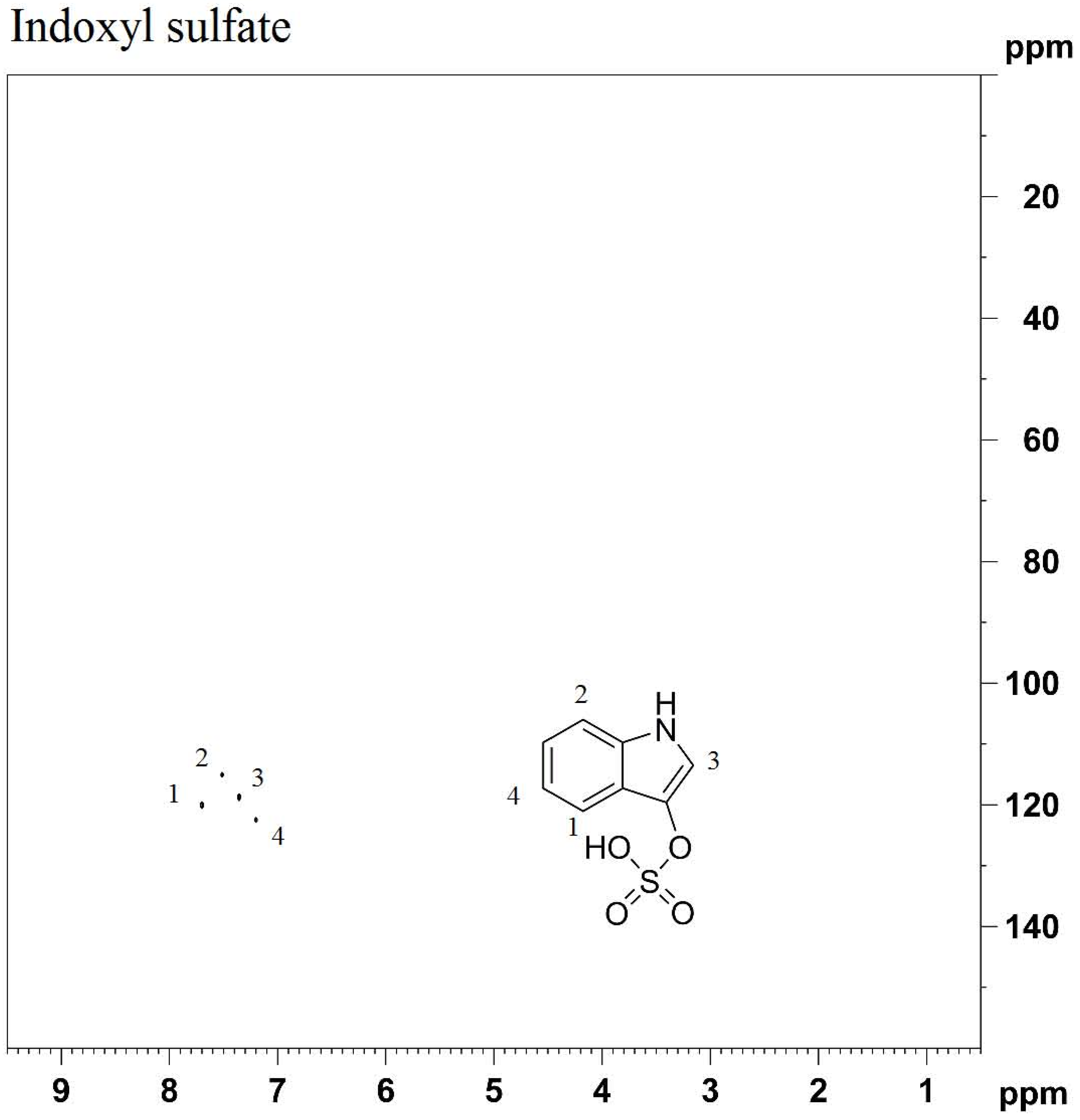


Phenylacetylglutamine

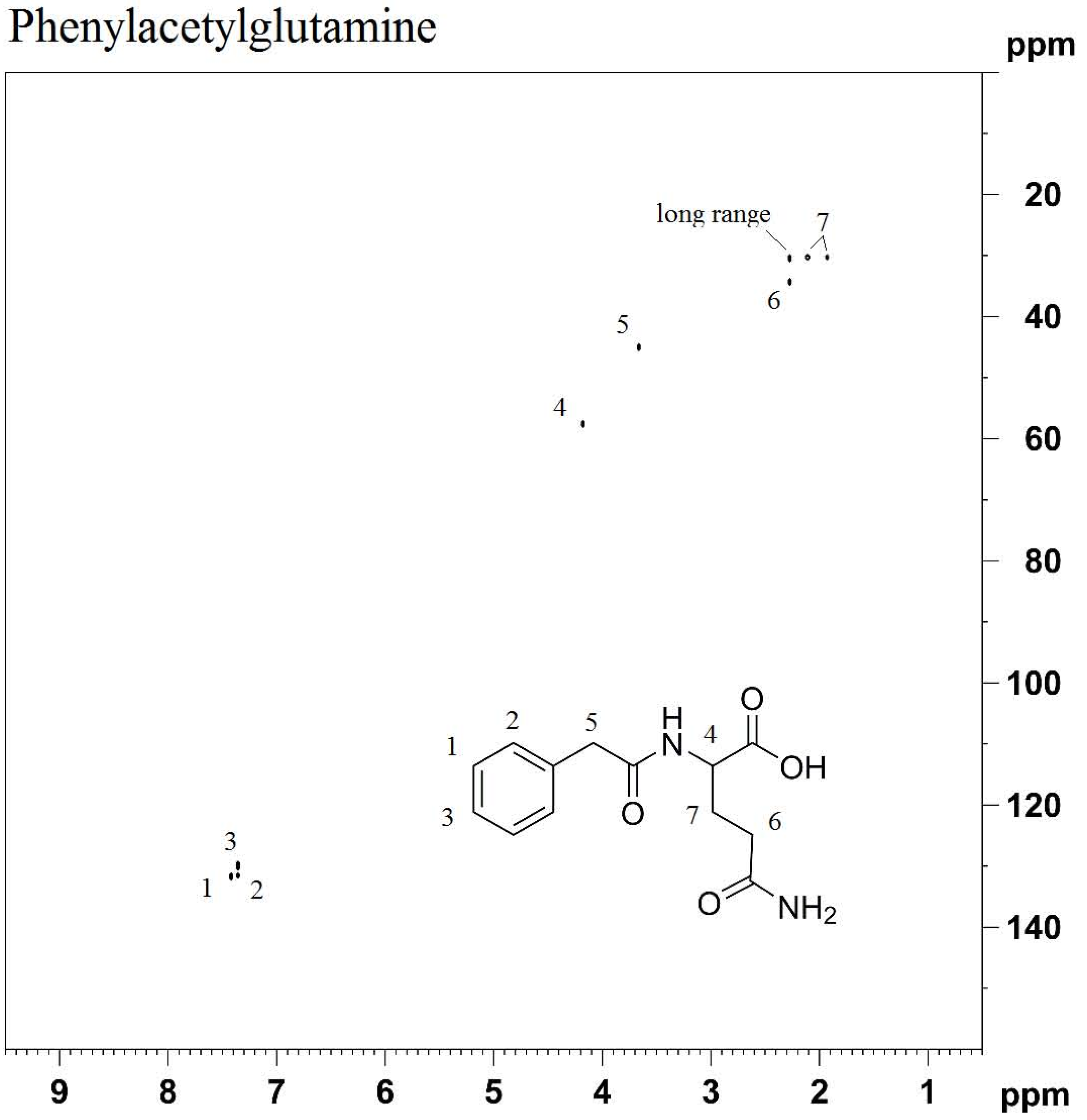


*trans*-Aconitic acid

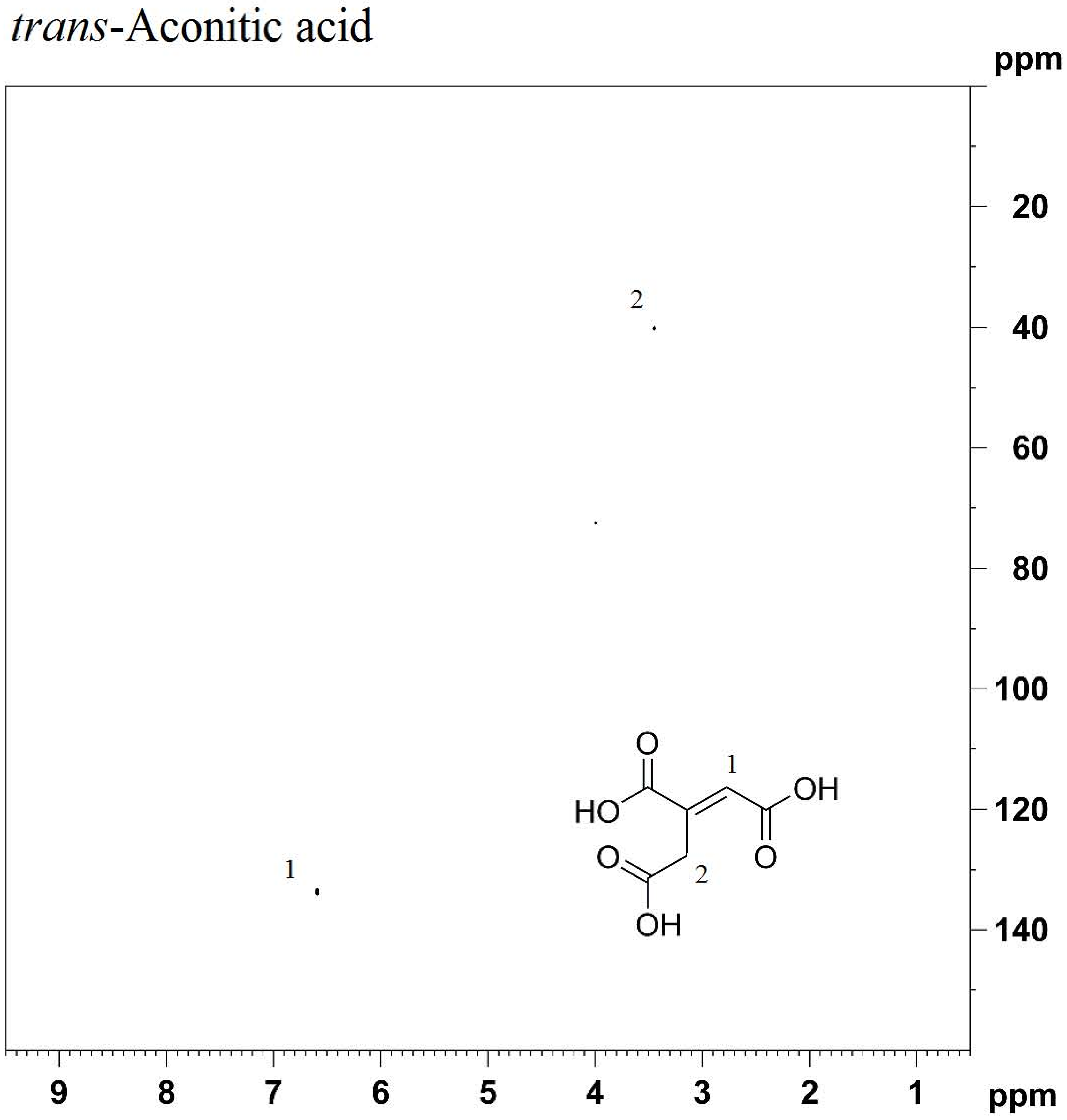


Levoglucosan

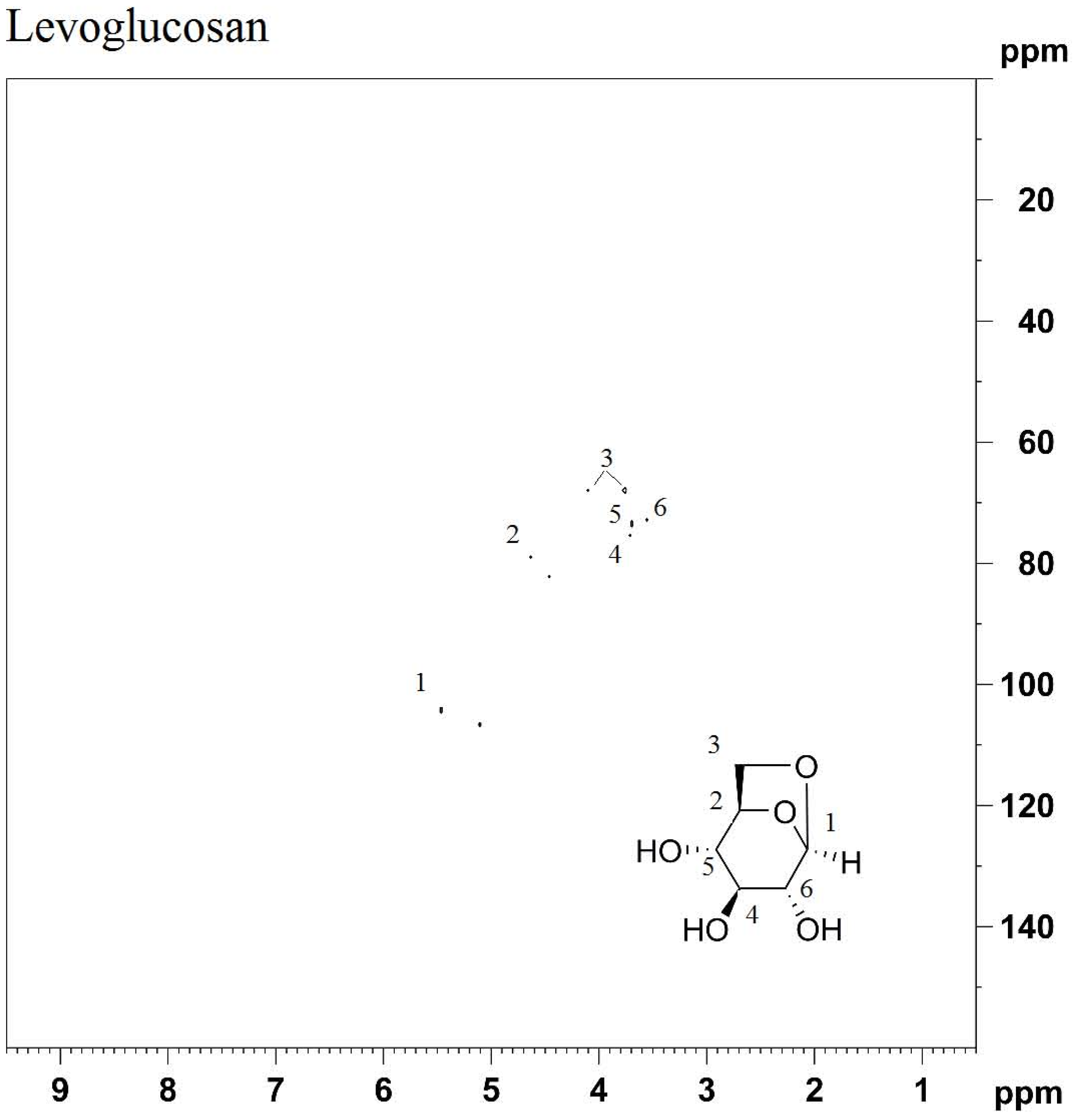

Carnitine

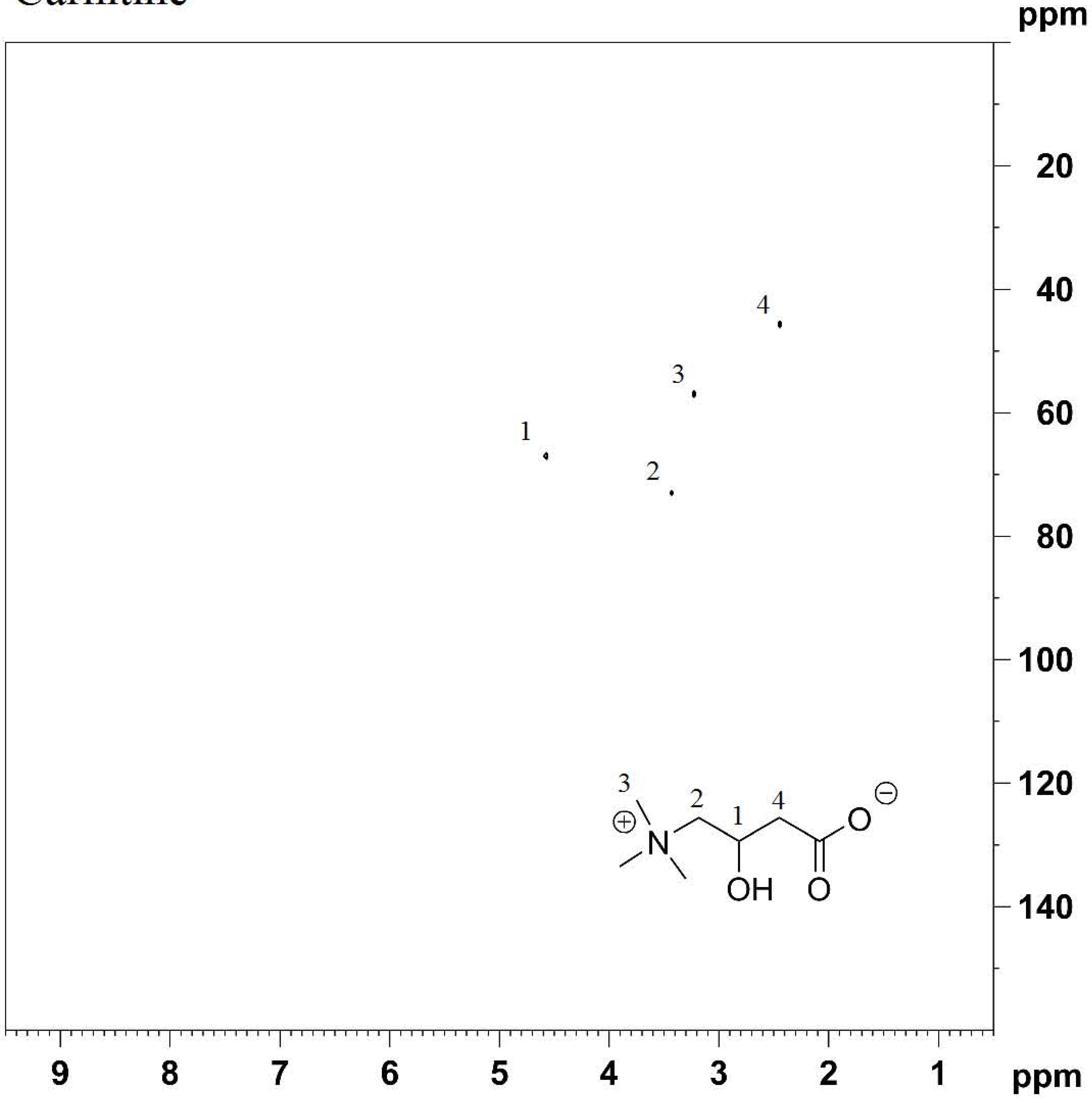


Creatine

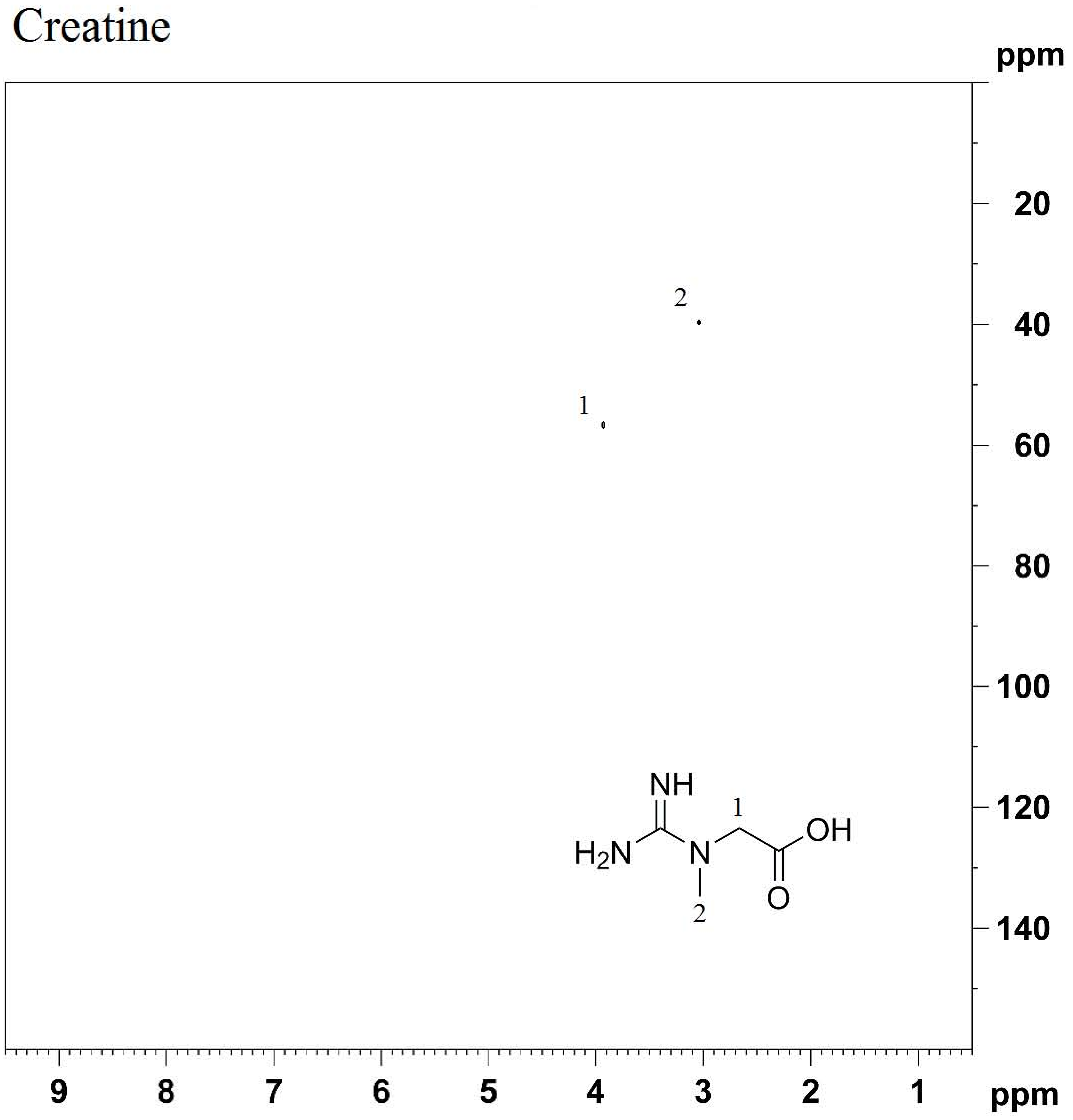


Mannitol

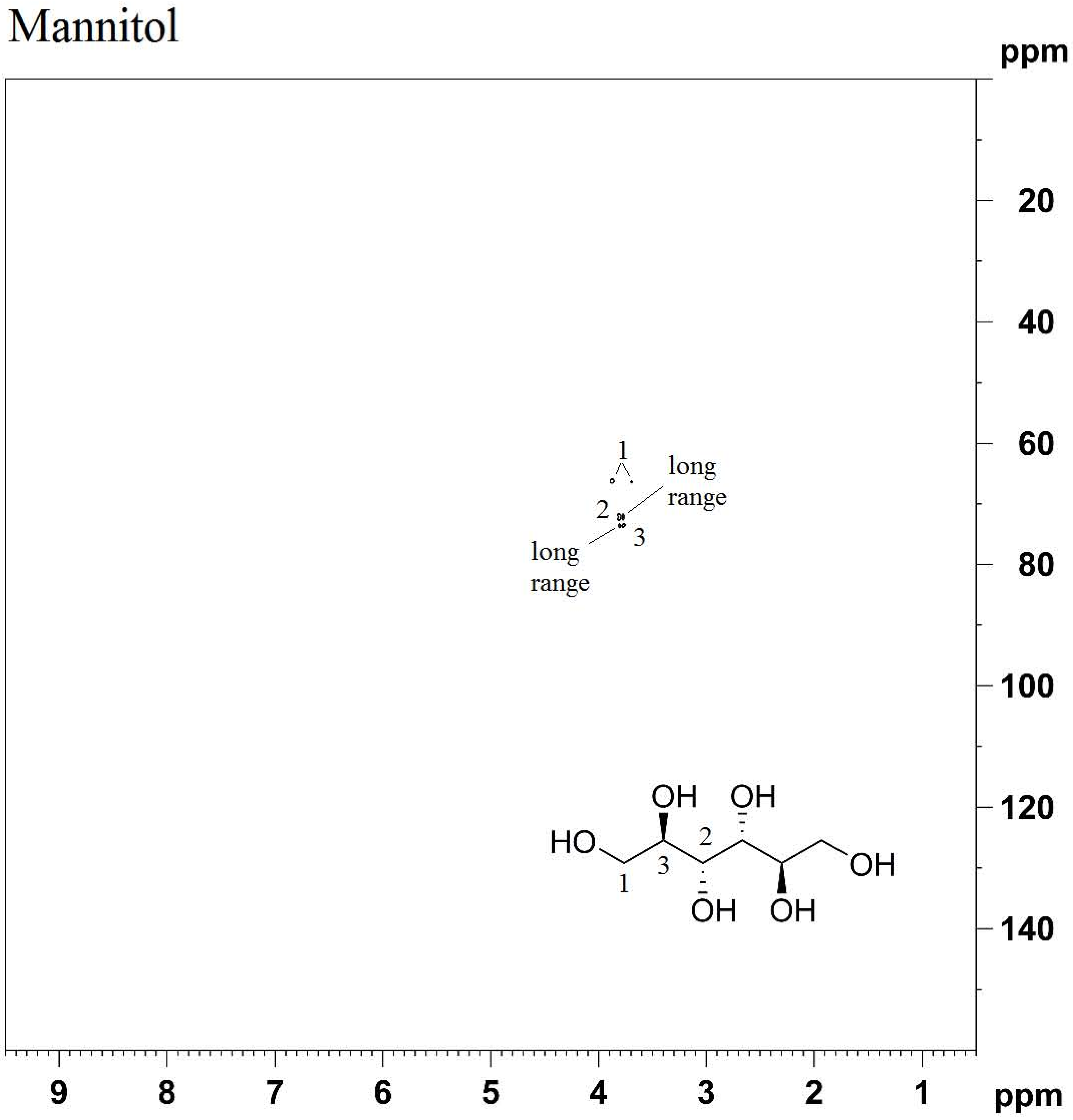


Erythritol

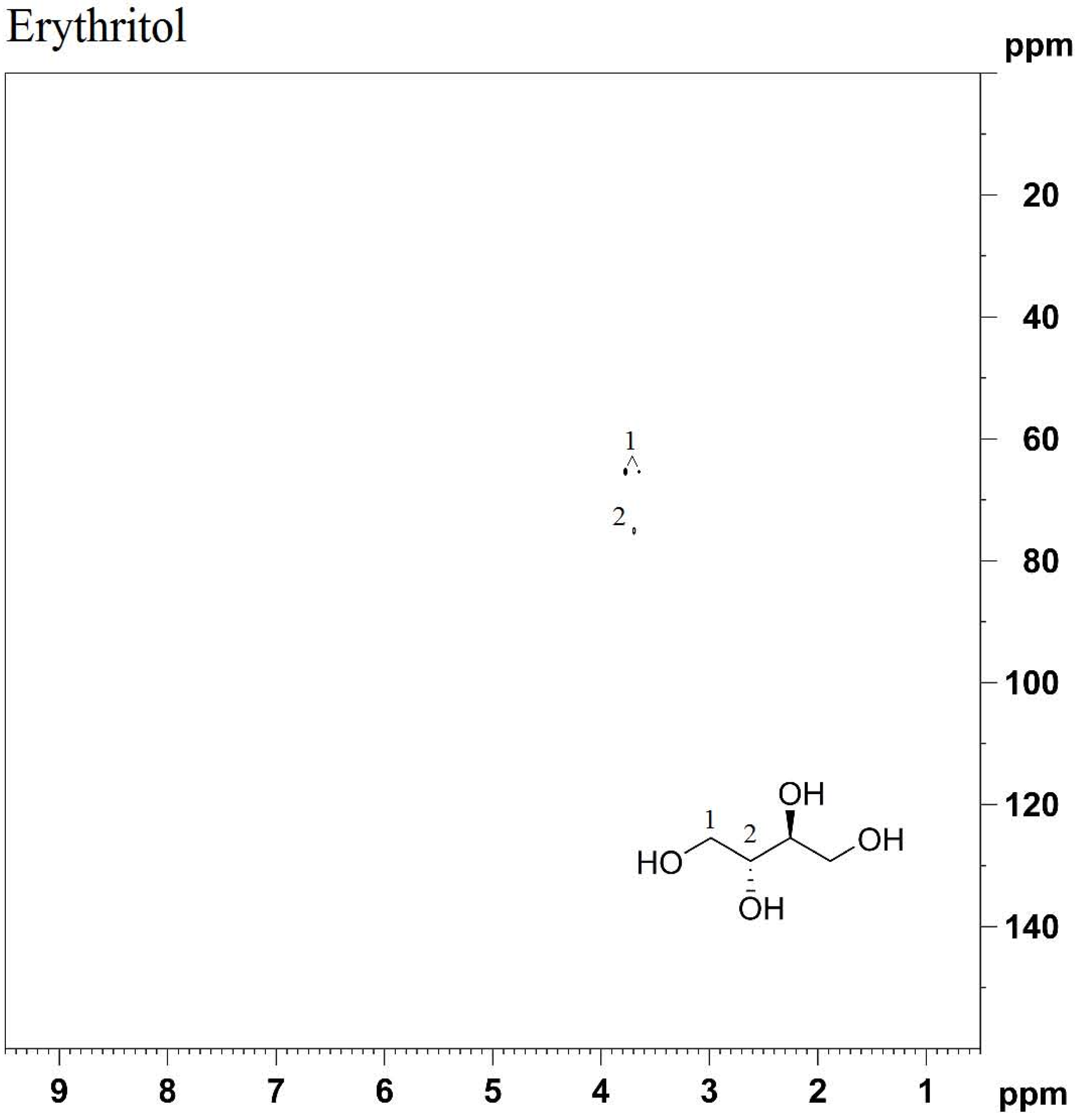


Galactitol

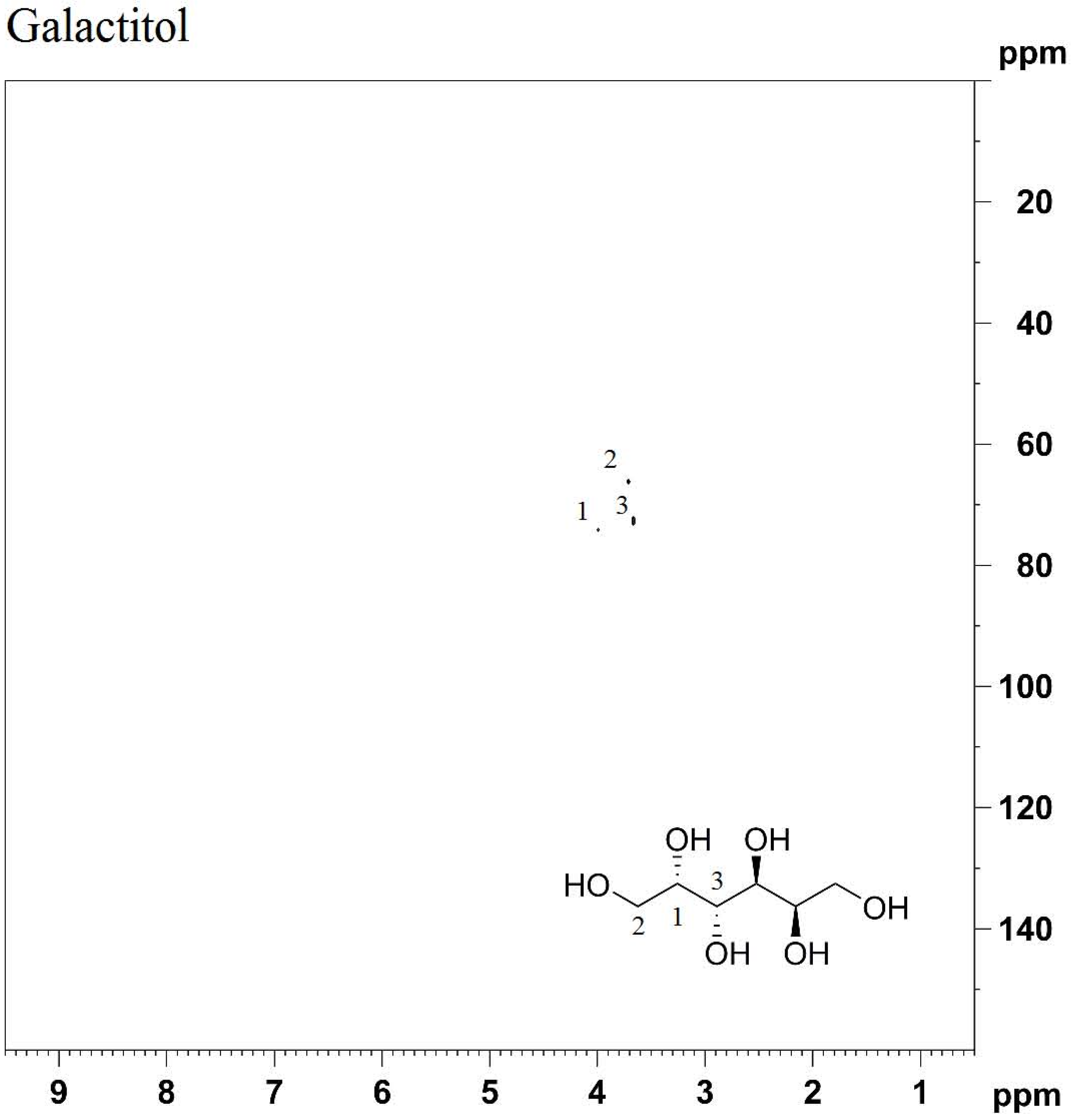


Taurine

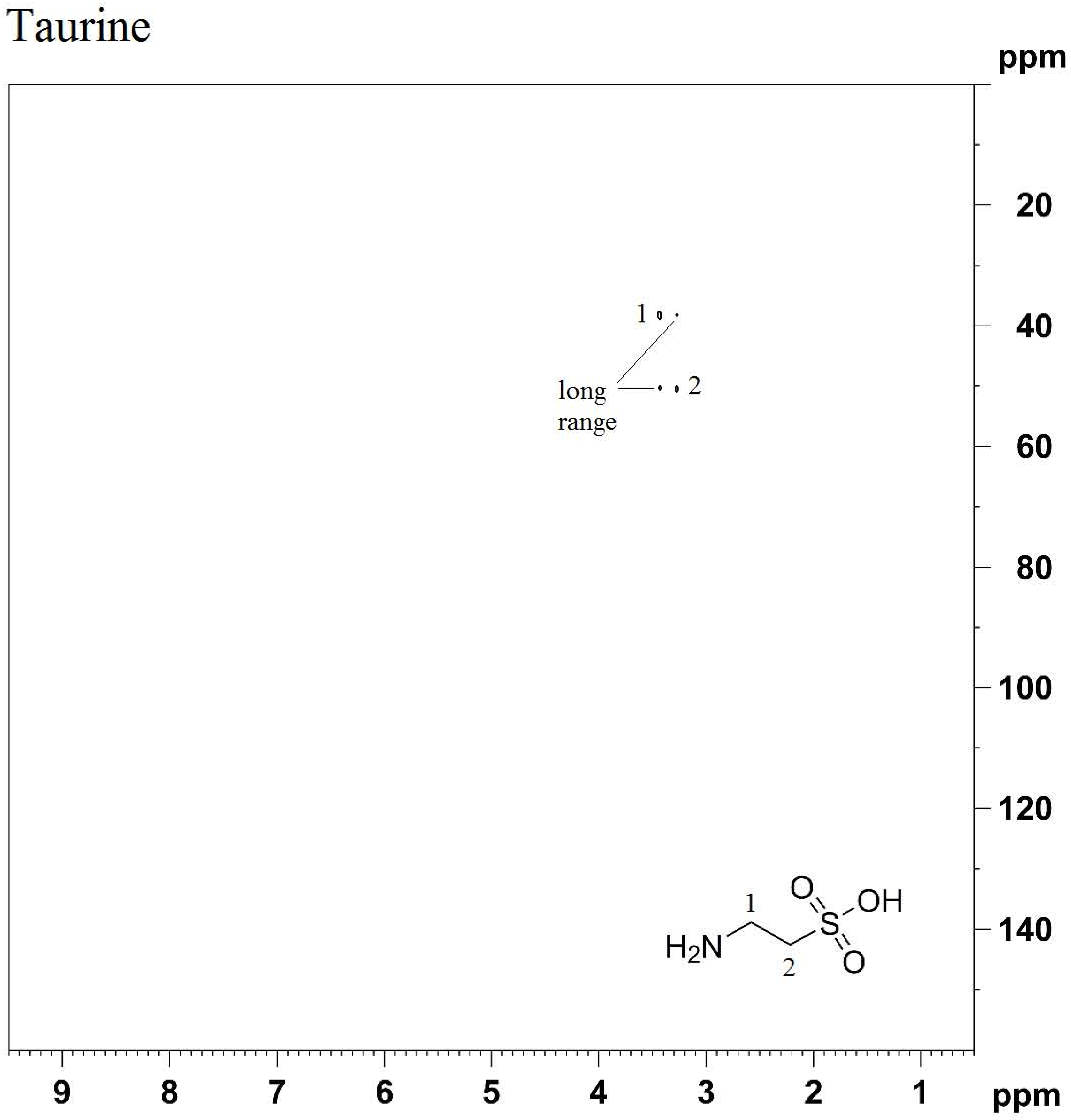

4-Hydroxyphenylacetic acid

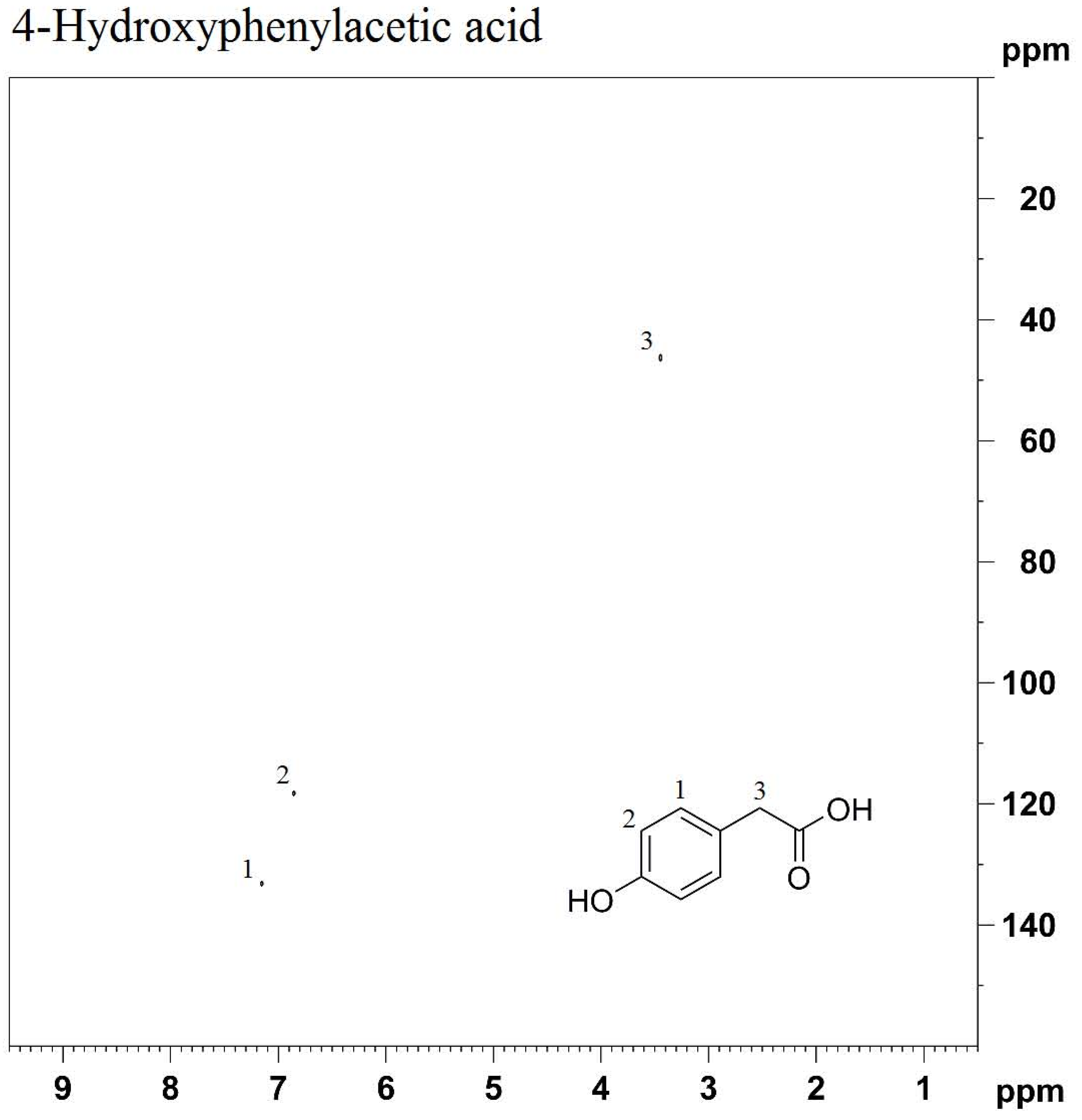


Betaine

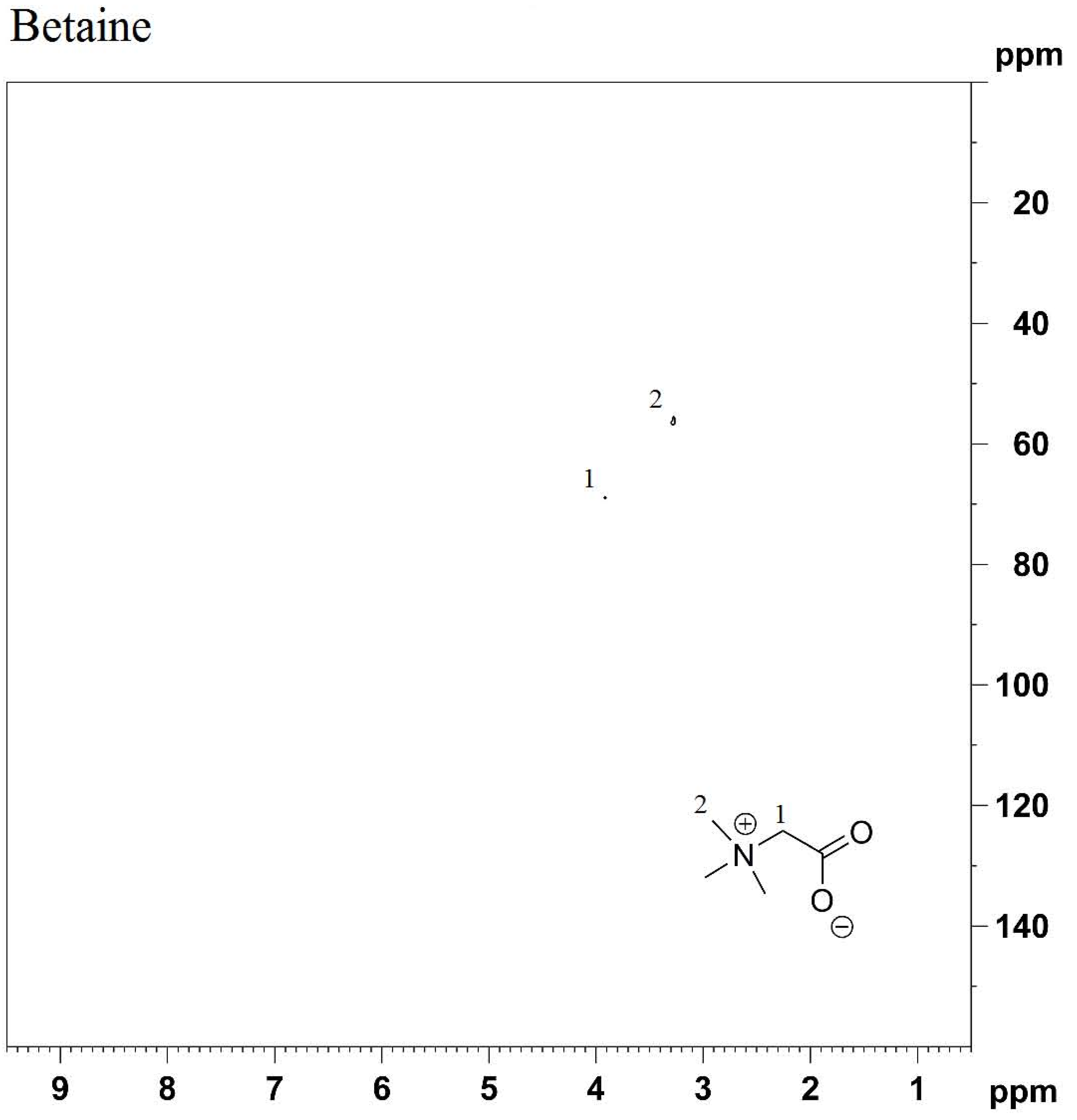


Ethanolamine

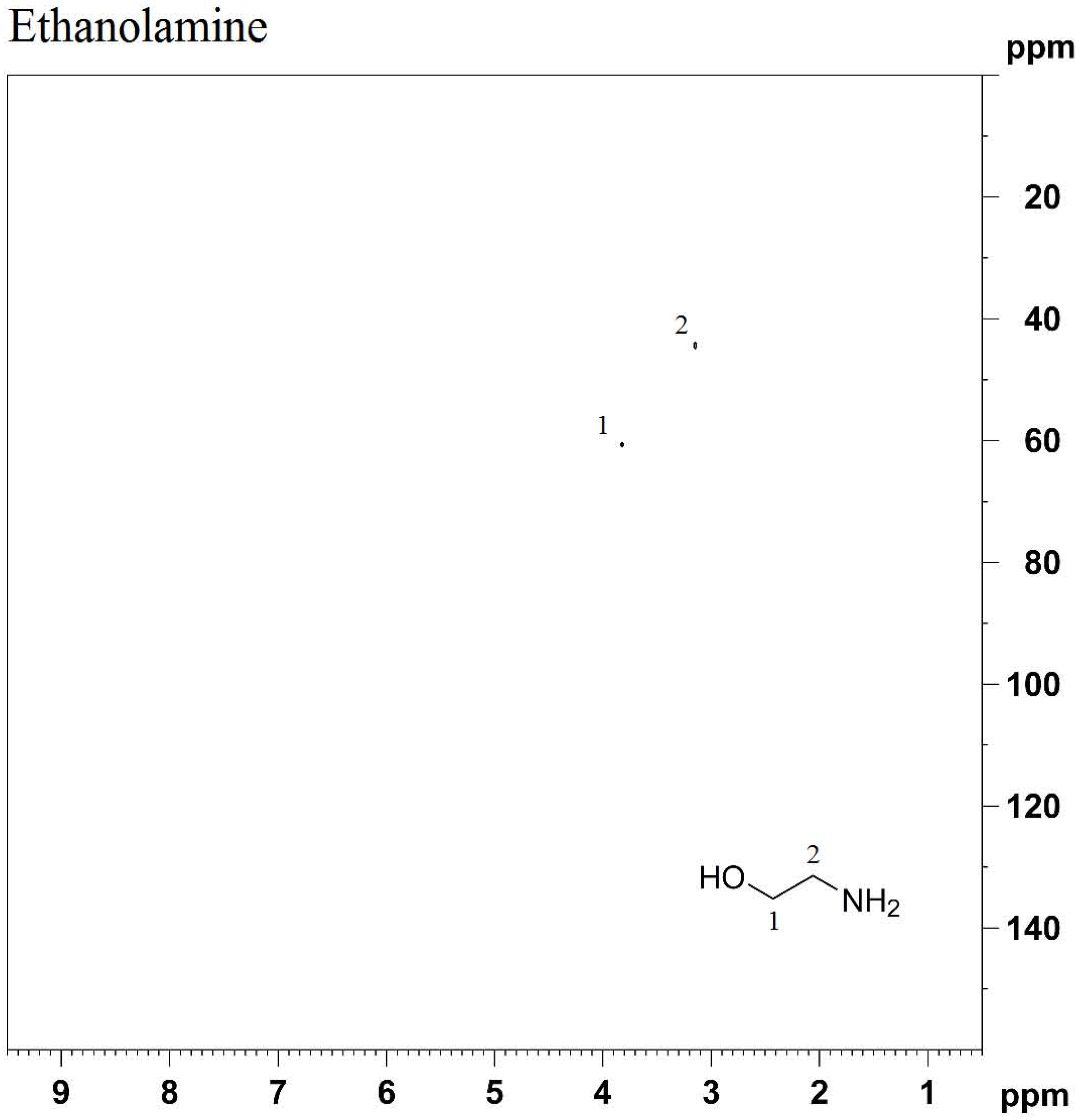


Isocitric acid

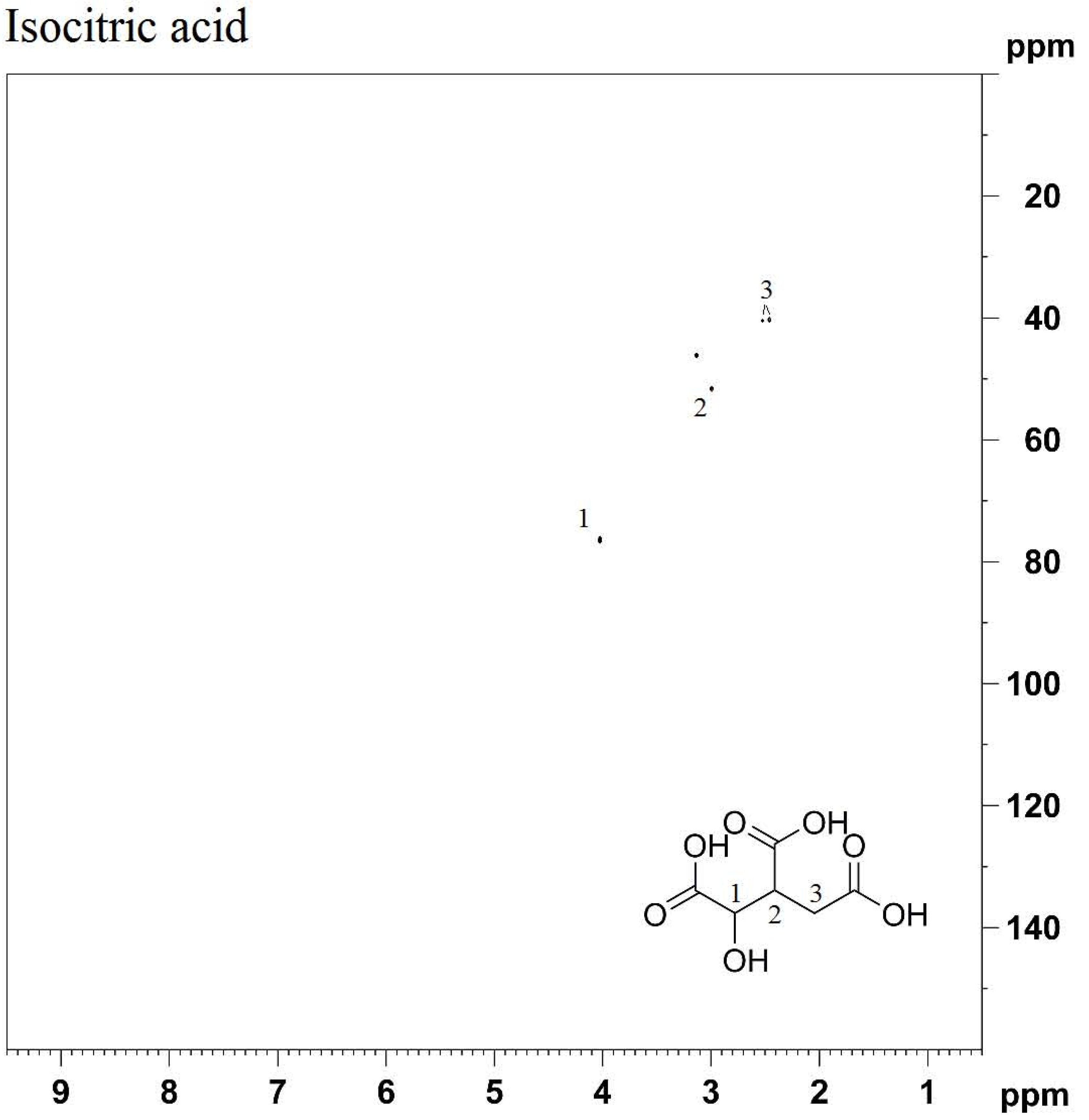


Citric acid

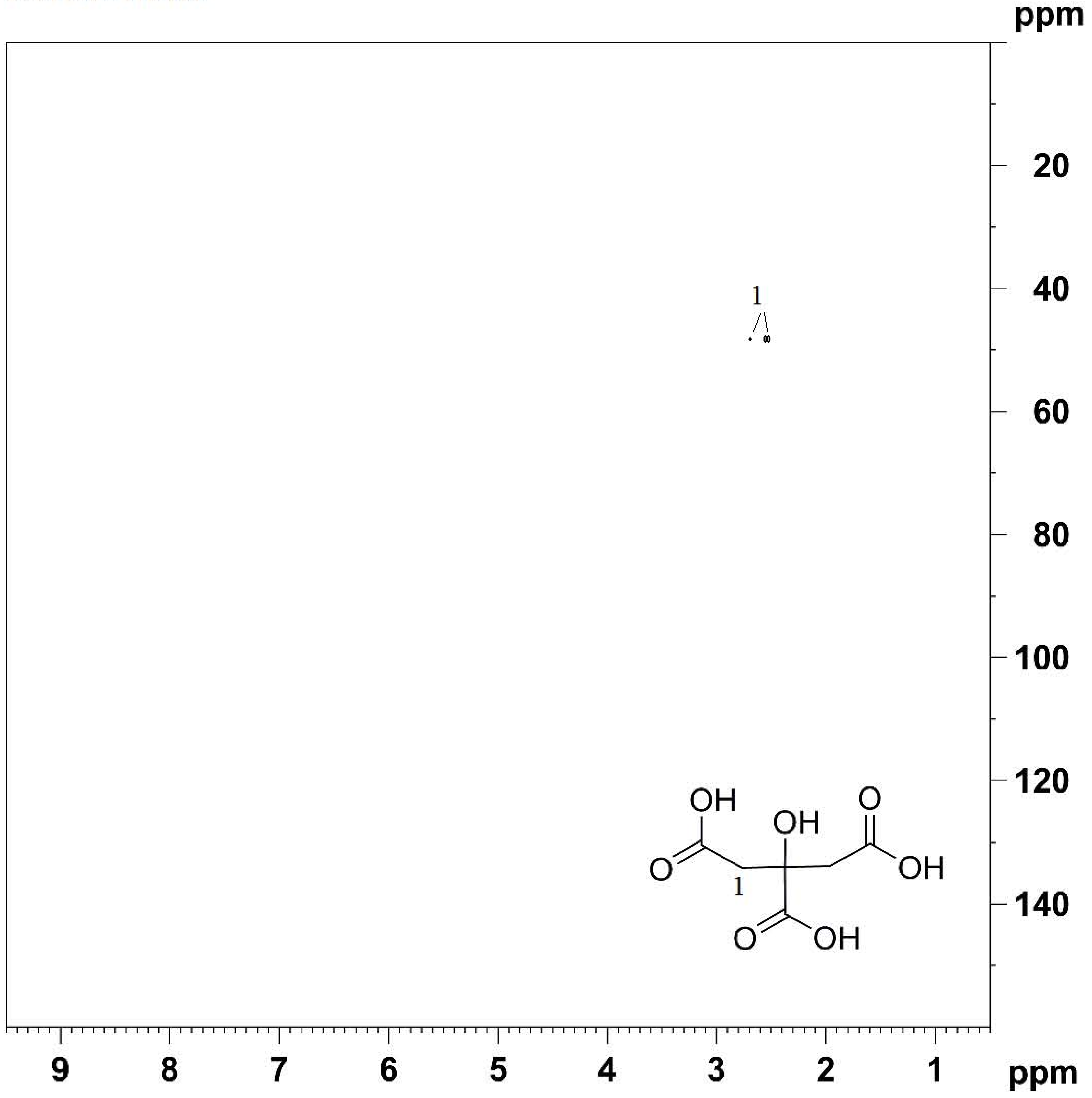


Glutamine

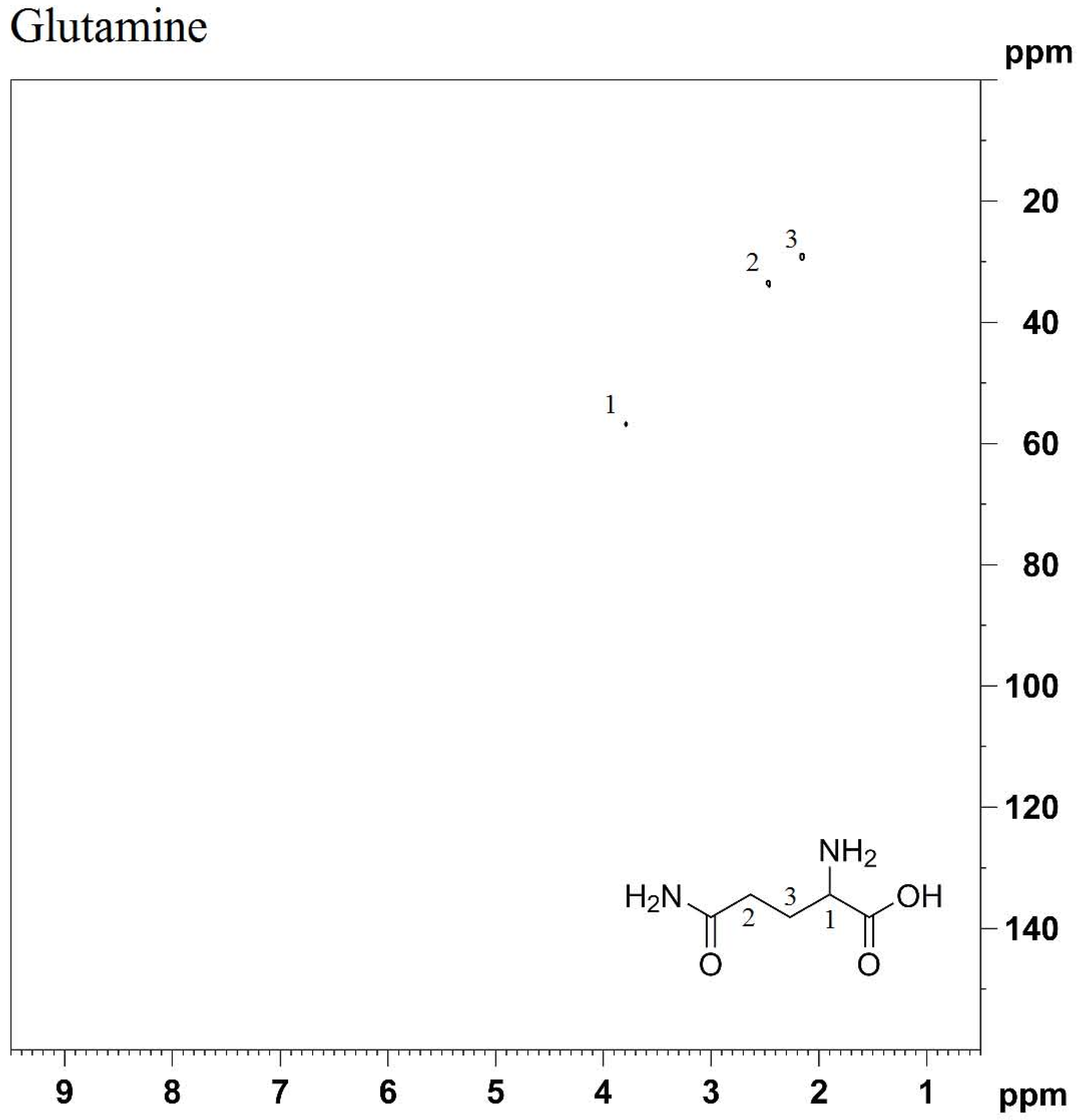

Spermine

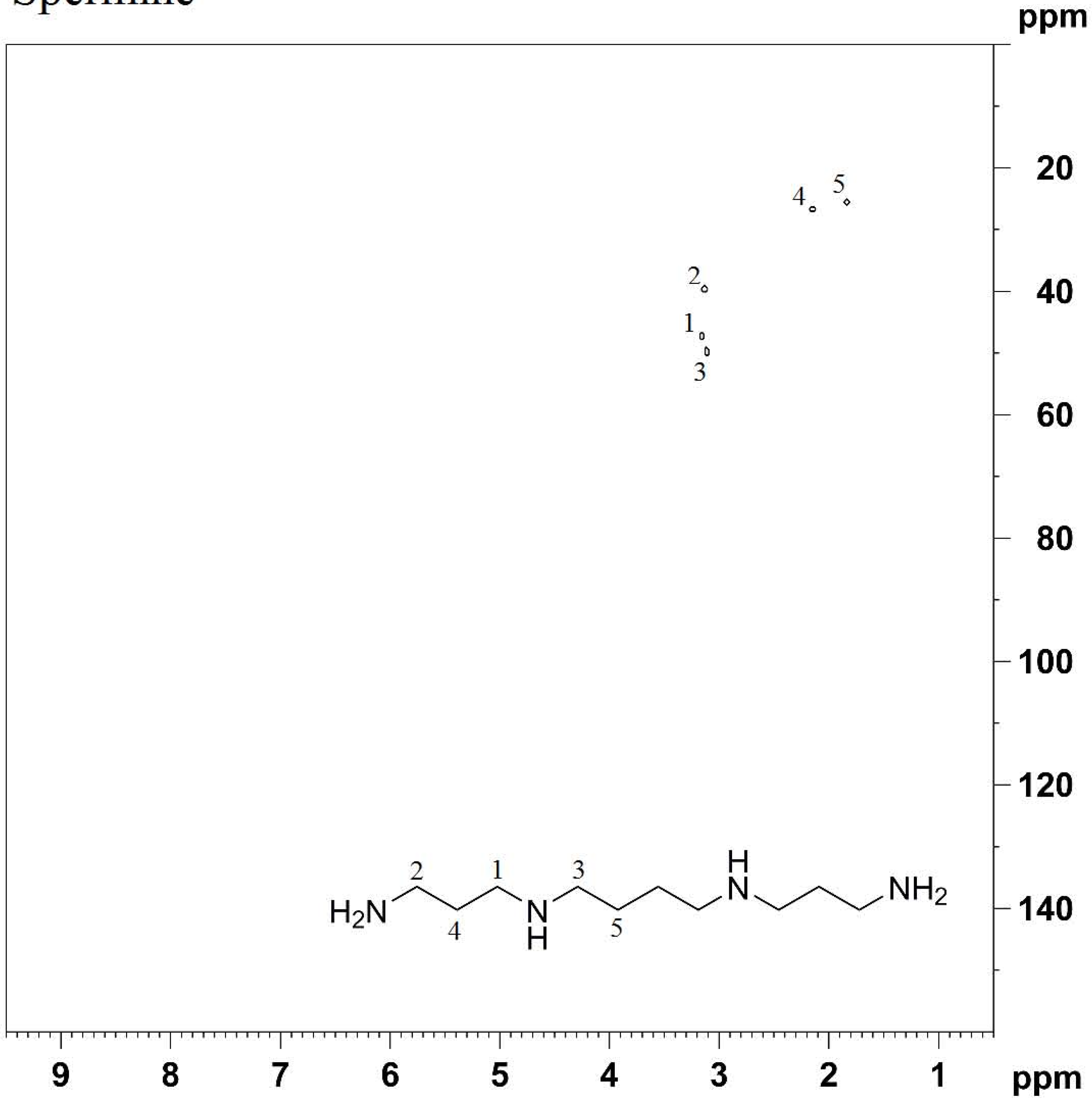


Lysine

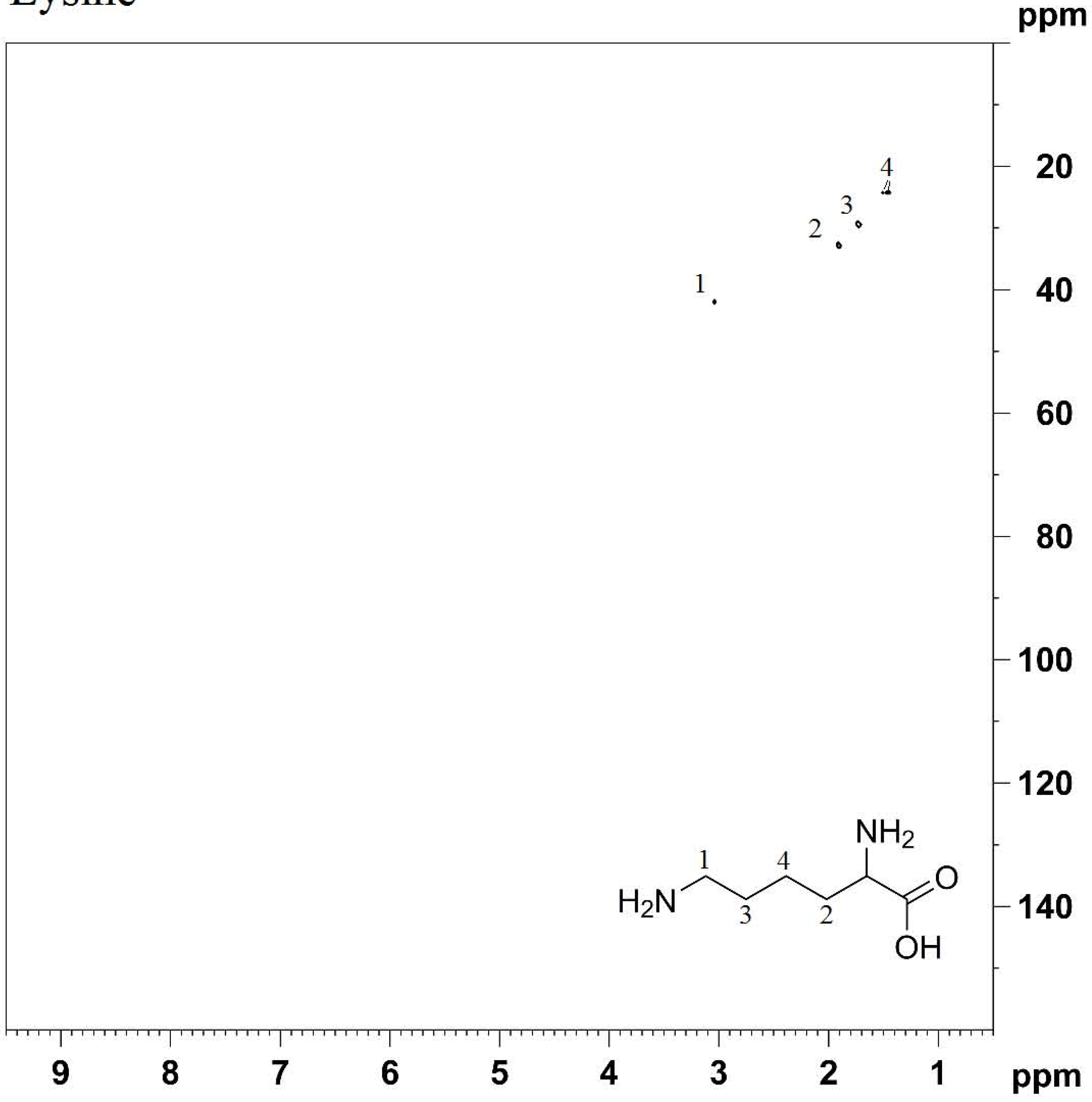


Adipic acid

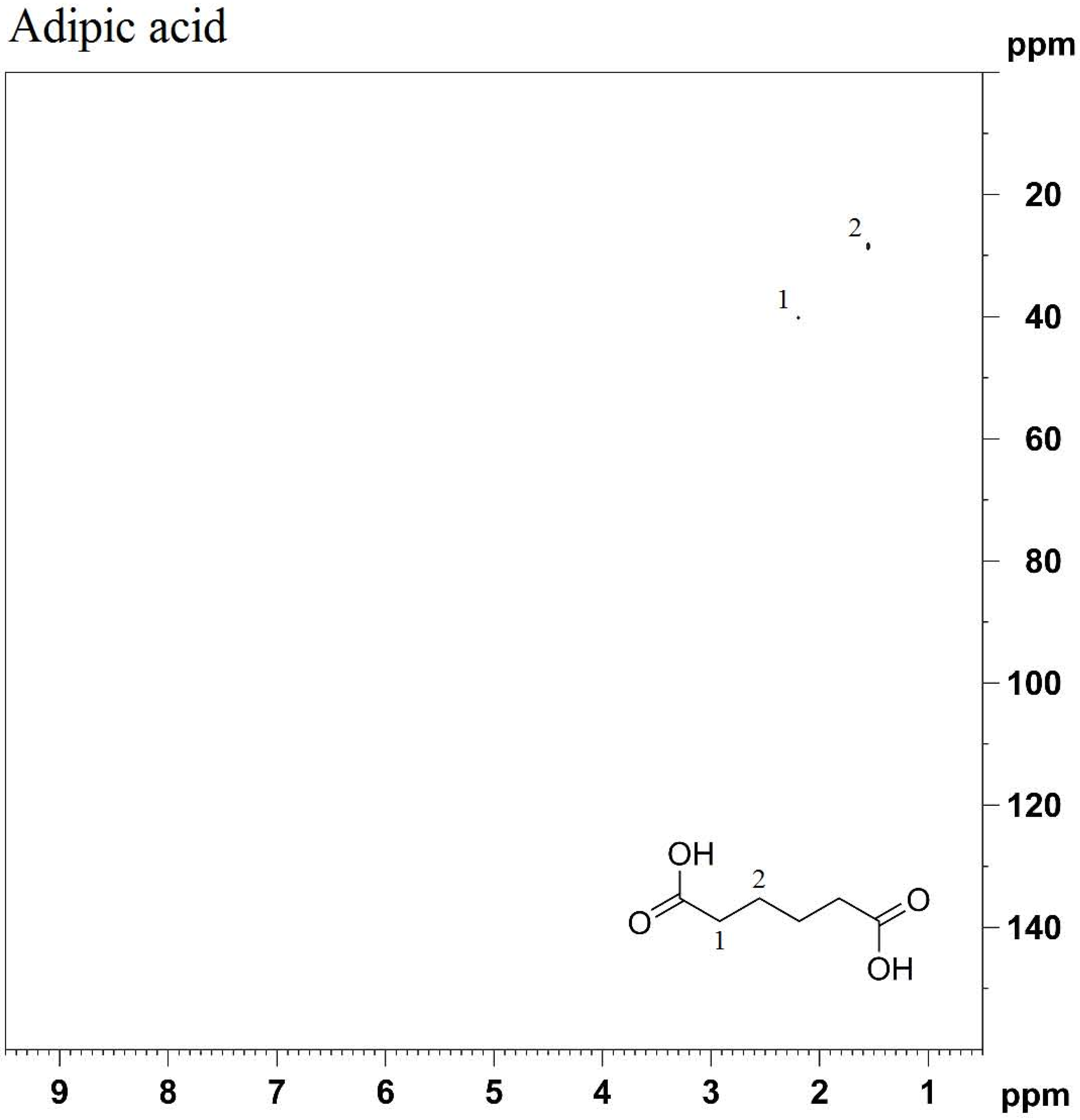


3-Hydroxyisovaleric acid

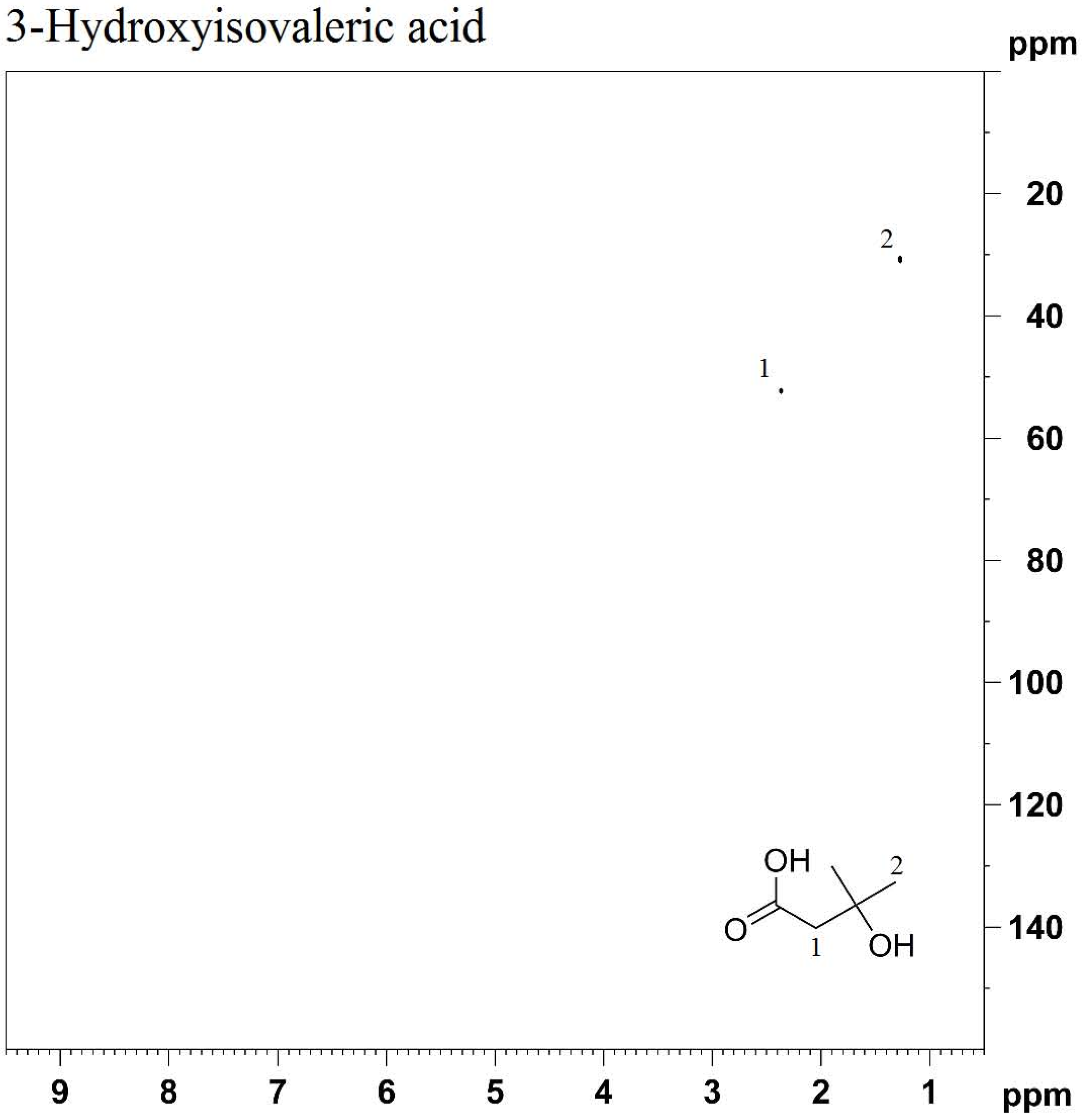


3-Aminoisobutanoic acid

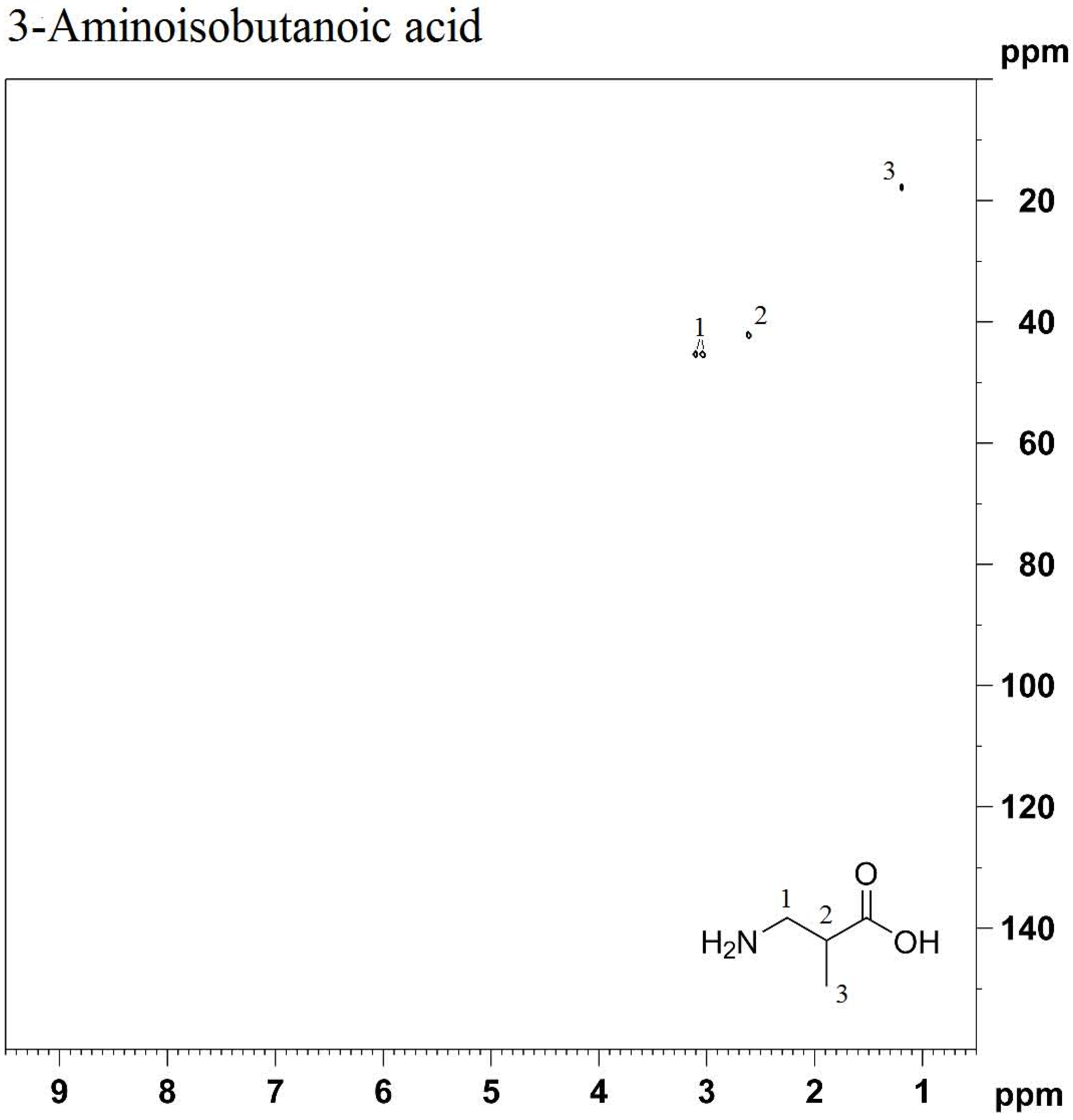

Supplement: Additional file 1: — Correlation_plots. Shows individual correlation plots from all 23 metabolites. [file 12859_2014_413_MOESM1_ESM.pdf]
